# Mapping the Global Landscape of Vaccine Acceptance Among the General Population from 2009 to 2024: A Systematic Review Across COVID-19 Pandemic Phases, Vaccine Categories, and Economic Strata

**DOI:** 10.3390/vaccines14080663

**Published:** 2026-07-29

**Authors:** Madan Khatiwada, Yu-Tan Chen, Carine Dochez, Peter Delputte

**Affiliations:** 1 Department of Biomedical Sciences, Universiteit Antwerpen, 2610 Antwerpen, Belgium; 2 Department of Public Health, Institute of Tropical Medicine, 2000 Antwerpen, Belgium; ychen@itg.be (Y.-T.C.); cdochez@itg.be (C.D.); 3 Laboratory for Microbiology, Parasitology and Hygiene (LMPH), Infla-Med Centre of Excellence, Department of Biomedical Sciences, Universiteit Antwerpen, 2610 Antwerpen, Belgium; peter.delputte@uantwerpen.be

**Keywords:** vaccination, vaccine acceptance, trends, time-period, COVID-19

## Abstract

**Background**: Vaccine acceptance is a fundamental prerequisite for uptake, which may vary over time in response to contextual and vaccine-specific factors and major global disruption. This systematic review and meta-analysis evaluated global trends and determinants of vaccine acceptance between 2009 and 2024. **Methods**: A comprehensive literature search was conducted using pre-defined keywords and controlled vocabulary related to vaccination, vaccine acceptance or hesitancy, with a broad search strategy designed to capture global evidence across multiple vaccine categories and geographic settings. Studies restricted to high-risk groups or other specific sub-populations (e.g., healthcare workers) were excluded. Studies reporting vaccine acceptance outcomes were systematically identified and pooled estimates were calculated across vaccine categories, WHO regions, income groups, and pandemic periods. Temporal trends and interaction effects were assessed to evaluate changes in vaccine acceptance among different vaccines and before, during, and after the COVID-19 pandemic. **Results**: A total of 264 publications, including 650,772 participants from 97 countries, were included. Overall, pooled vaccine acceptance was 70.63% and remained relatively stable throughout the study period. Most studies originated from high-income countries, whereas low-income countries and the African region were underrepresented. Childhood vaccines demonstrated the highest pooled acceptance (80.26%), followed by HPV vaccines (66.48%), while influenza vaccines showed the lowest acceptance (52.00%). Childhood vaccine acceptance increased over time and during the COVID-19 period, whereas significant negative interaction trends were observed for HPV and influenza vaccines. Higher vaccine acceptance was observed in African and South-East Asian regions compared with Western Pacific and European regions. Acceptance also varied across income groups, with greater hesitancy generally observed in higher-income settings. **Conclusions**: Vaccine acceptance among the general population remained relatively stable globally between 2009 and 2024 but varied substantially by vaccine categories, geographic regions, and economic settings. These findings highlight the context-specific nature of vaccine acceptance and the need for harmonized measurement tools and stronger evidence from underrepresented regions to support equitable immunization strategies.

## 1. Introduction

Vaccination remains one of the most effective public health interventions, preventing millions of deaths annually [1]. It was essential for the eradication of smallpox and brought diseases such as polio close to eradication [1]. As immunization coverage expands, the perceived importance of vaccination may decline as public awareness of disease risks diminishes. In such contexts, adverse events (perceived, real or potential) associated with vaccines can attract more attention than the diseases themselves. Despite the proven safety, efficacy, and success of vaccines, challenges related to overall vaccine acceptance persist as major global public health concerns [2,3,4].

**Figure 1:**
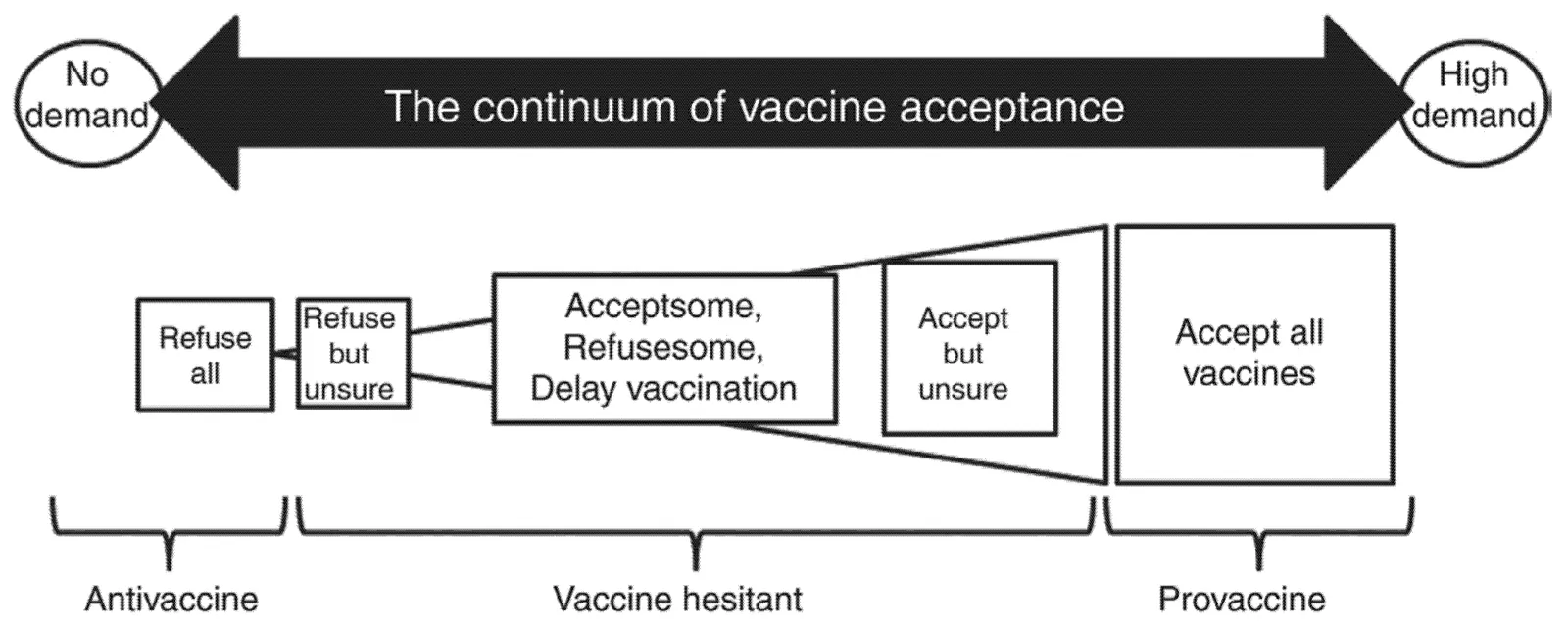
The continuum of vaccine acceptance [5].

Vaccine acceptance, defined as the willingness to receive vaccination, is a critical determinant of public health outcomes and is a complex interplay of factors often specific to the context and community, and can vary across time, place, disease, and type/category of vaccine [5,6,7]. For example, individuals may accept routine immunizations for their children but be hesitant to seek COVID-19 or flu vaccination for their child or themselves [8]. Vaccine acceptance exists on a continuum, between full acceptance and outright refusal of all vaccines. Individuals may occupy various positions along the continuum of attitudes and behaviors, with these positions subject to change depending on temporal, contextual and vaccine-specific factors (Figure 1) [5]. Vaccine hesitancy is a concept which is now frequently used in discussions of vaccine acceptance and is influenced by a number of factors including issues of confidence (do not trust vaccine or provider), complacency (do not perceive a need for a vaccine, do not value the vaccine), and convenience (access) [5,9,10].

Vaccine acceptance/hesitancy is influenced by multiple factors, including individual beliefs, sociocultural influences, and the foundation of healthcare systems and governments. Over the past decades, vaccine acceptance has fluctuated due to various factors, including public trust in health authorities, various global events, including pandemics, misinformation campaigns, social media platforms, public health policies and perceived vaccine safety [11,12].

The period from 2009 to 2024 encompasses significant events that have influenced public perception of vaccines. The 2009 H1N1 influenza pandemic, for instance, was a pivotal moment that affected vaccine acceptance. A study analyzing data from the French general population revealed that vaccination acceptability decreased from 91.1% in 2000 to 61.2% in 2010, coinciding with the H1N1 pandemic [13]. This decline was attributed to factors such as public skepticism towards the rapid development and distribution of the H1N1 vaccine, as well as concerns about its safety and efficacy. In addition, the global expansion of social media platforms such as Facebook has amplified the intensity and nature of the information shared, thereby increasing vulnerability to vaccine- and vaccination-related misinformation and disinformation [14,15]. In the subsequent years, efforts to improve vaccine acceptance faced more challenges, including the spread of misinformation and the rise of the anti-vaccine movement. The proliferation of false information, particularly through social media platforms, has been identified as a significant barrier to vaccine uptake. For example, conspiracy theories linking the measles, mumps, and rubella (MMR) vaccine to autism have persisted despite being thoroughly debunked, thereby continuing to contribute to vaccine hesitancy [16]. In 2019, the United States experienced 1249 reported cases of measles—the highest annual incidence since 1992—largely attributed to vaccine hesitancy, particularly toward the measles vaccine. Notably, 89% of these cases involved individuals who were either unvaccinated or had unknown vaccination status, and 86% were linked to outbreaks within under-immunized, close-knit communities characterized by shared belief systems that discourage vaccination [17].

The COVID-19 pandemic further complicated the landscape of vaccine acceptance. While the development of COVID-19 vaccines was a scientific milestone, it also led to heightened public scrutiny and debate over vaccine safety and mandates. This period saw a polarization of opinions, with some individuals showing greater acceptance of vaccines due to the perceived threat of the virus, while others grew more skeptical, influenced by misinformation, political factors, and at times unclear communication from authorities. Government communication often reflected the incremental increase in scientific knowledge, requiring frequent adaptations as new evidence emerged, which sometimes contributed to confusion and mistrust among the public. Notably, the anti-vaccine movement gained political traction during this time, with some prominent politicians/political affiliates advocating for re-evaluation of vaccination schedules, thereby amplifying vaccine hesitancy [15,18,19,20].

This systematic review synthesizes evidence on how vaccine acceptance, hesitancy, intention to vaccinate, and trust in vaccines have evolved globally from 2009 to 2024, and the impact of the COVID-19 pandemic on vaccine acceptance. The review also identified key trends of vaccine acceptance across time periods (before, during, and after the COVID-19 pandemic), vaccine categories and country income levels.

## 2. Methodology

### 2.1. Protocol Registration and Reporting Standards

The systematic review protocol was registered in the International Prospective Register of Systematic Reviews (PROSPERO) (registration number: CRD42024607828). The registered protocol predefined objectives, eligibility criteria, screening procedures, data extraction and analytical approaches.

This review was conducted and reported in accordance with the Preferred Reporting Items for Systematic Reviews and Meta-Analyses (PRISMA 2020) statement [21]. The PRISMA checklist is available as Supplementary Materials (File S1: PRISMA checklist).

### 2.2. Eligibility Criteria

Eligibility criteria were defined using a population-exposure-outcome-study design framework.

#### 2.2.1. Population

The review focused exclusively on studies conducted among the general public. Studies were excluded if they targeted specific subpopulations that are not representative of the general population, including: Healthcare workers (e.g., physicians, nurses, midwives, etc.), Individuals with specific diseases or health conditions (e.g., diabetes, cardiovascular disease, pregnancy, etc.), Minority or special populations (e.g., immigrants, prisoners, asylum seekers, etc.), Specific professional or occupational groups (e.g., university students, construction workers).

#### 2.2.2. Vaccines of Interest

The study focused on acceptance of vaccines that are globally utilized, including general childhood vaccines (Measles, mumps, and rubella (MMR/MR/measles)), Hepatitis B, Diphtheria, Tetanus, and Pertussis, Rotavirus, *Haemophilus influenzae* type b, Pneumococcal conjugate vaccines, oral or inactivated polio vaccines, acceptance of influenza vaccines in elderly, adult and children, and acceptance of human papillomavirus (HPV) vaccines for adolescents. Since the main interest of the study was vaccine acceptance, studies that did not mention specific vaccines but instead assessed the attitude to vaccines in general were included.

Studies that evaluated specific vaccines were excluded if they met any of the following criteria: Pandemic-specific vaccines (e.g., 2009 H1N1 influenza vaccine, COVID-19 vaccines), Vaccines introduced during the study period (e.g., RSV vaccines), Vaccines not routinely used in more than 50% of countries globally at the start of the COVID-19 pandemic (e.g., varicella, rabies), vaccines targeting specific regions (e.g., cholera, typhoid, Japanese encephalitis, meningococcal, yellow fever), Vaccines mainly used in the high-income settings (e.g., zoster vaccine).

#### 2.2.3. Study Designs

Included study designs were: Cross-sectional studies, Survey-based studies, Cohort studies reporting baseline or observational acceptance outcomes.

Excluded study designs were: Clinical trials, Intervention studies, Case–control studies, Vaccine effectiveness or immunogenicity studies.

#### 2.2.4. Time Frame and Language

Eligible studies were those conducted between 1 January 2009 and 31 December 2024, regardless of publication year, provided that data collection occurred within this period. Only studies published in English were included.

### 2.3. Information Sources and Search Strategy

A comprehensive literature search was conducted across different electronic databases (PubMed, Embase, Scopus and Cochrane Library) using predefined keywords and controlled vocabulary related to vaccination, vaccine acceptance, hesitancy, confidence, trust, and public attitudes. The search strategy was intentionally broad to capture global evidence spanning multiple vaccines and geographic contexts. The search strings used are included in File S2: Database Keyword Search. The search was run across the databases during the period of 01–10 April 2025. Records retrieved from all databases were combined and duplicate records identified across and within databases were removed prior to screening to ensure that each unique record was considered only once and to prevent duplication of evidence during study selection and synthesis.

### 2.4. Study Selection Process

Study selection was conducted in three sequential stages: (a) All the articles retrieved were first screened by title to remove clearly irrelevant records after removing duplicate records. (b) The articles were then screened by title and abstract separately by two reviewers as per the defined inclusion and exclusion criteria. (c) The retained articles were assessed through full-text review to determine final inclusion.

During the process (b) and (c), if there was any disagreement between the reviewers on whether the study should be included, the two reviewers discussed together and reached the final agreement.

**Figure 2:**
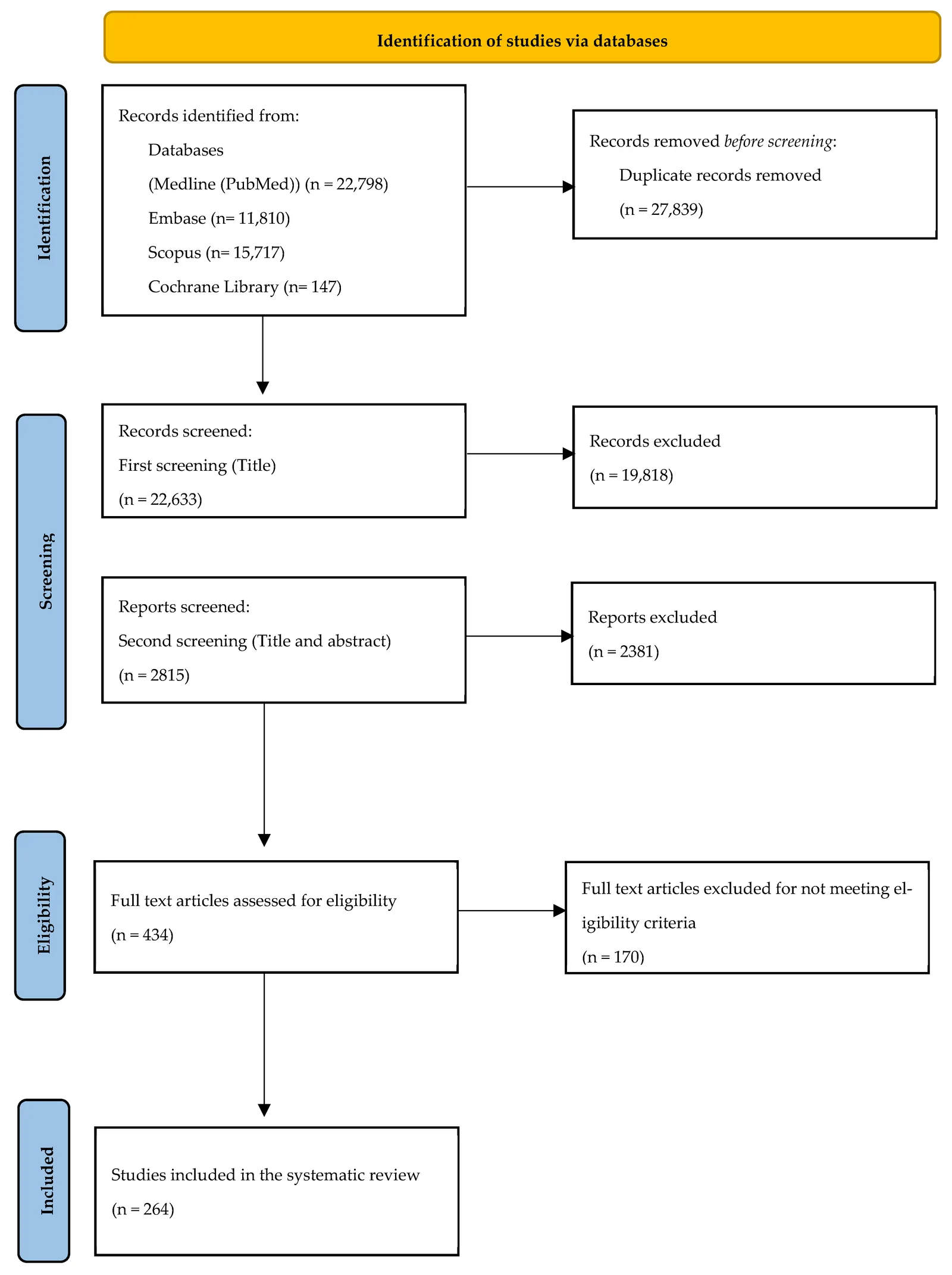
PRISMA 2020 flow diagram for systematic review on vaccine acceptance from 2009 to 2024.

The full selection process is summarized in the PRISMA flow diagram (Figure 2).

### 2.5. Data Extraction

A standardized data extraction form was developed and pilot-tested prior to full extraction. Two reviewers independently extracted data from all included studies to minimize error and bias. Extracted variables included:

*Study Characteristics:* Author(s), Year of publication, Country, Study period (start and end dates), Study design and Sample size.

*Population Characteristics:* Age group(s), classified according to World Health Organization (WHO) age categories—Children (0–9 years), Adolescents (10–19 years), Adults (20–64 years) and Older adults (≥65 years).

*Vaccine-Related Outcomes and Attitudes:* Vaccine category (general vaccine attitude, routine childhood, influenza, HPV, or other eligible vaccine), any vaccine acceptance or hesitancy metrics (including but not limited to the wording such as intention, decided to vaccinate, interested, willingness, reluctance, negative or positive attitude…).

*Country Income Classification:* Countries were classified according to the World Bank income classification applicable at the time of study publication: Low-income countries, Lower-middle-income countries, Upper-middle-income countries and High-income countries. We used the classification from 2024 to 2025. However, for countries that were labeled as “Unclassified” by the World Bank, the last available classification was used.

*Geographic region classification:* Countries were classified according to WHO regions applicable at the time of study publication: African (AFR), Americas (AMR), Eastern Mediterranean (EMR), European (EUR), South-East Asia (SEAR) and Western Pacific (WPR). Countries included in this review but not listed in WHO (Hong Kong, Taiwan and State of Palestine) were grouped based on the geographical location; Hong Kong and Taiwan in WPRO and State of Palestine in EMRO.

### 2.6. Time Period Classification

To evaluate temporal trends and contextualize the impact of the COVID-19 pandemic, data collection dates were analyzed both as a continuous variable and as a tripartite categorical variable (Pre-COVID, During-COVID, and Post-COVID).

Studies which reported specific start and end dates for data collection, a temporal midpoint was calculated to represent the study’s timing. In cases where the study only specified the month and year of data collection, the start date was standardized to the first day of the starting month, and the end date was standardized to the last day of the concluding month. The average of these two dates was then used as the study midpoint. These study midpoints were used as a continuous variable in the analysis.

For analyzing time as a categorical factor, studies were stratified into three distinct phases based on the recorded studies’ initiation and ending dates and the established WHO milestones on the COVID-19 pandemic:

- Pre-COVID: Studies with a data collection end date prior to 11 March 2020, the date on which the WHO officially declared COVID-19 a global pandemic.
- Post-COVID: Studies with a data collection start date following 5 May 2023, the date on which the WHO declared the end of the COVID-19 global health emergency.
- During-COVID: Any study for which the data collection period overlapped with or was contained entirely within the window between the aforementioned dates (11 March 2020 to 5 May 2023).

### 2.7. Data Synthesis and Analysis

A systematic quantitative synthesis was conducted to analyze the collected data. Descriptive statistics, including absolute frequencies and percentages, were used to summarize the characteristics of all included studies and their respective data points. These summaries encompassed vaccine target populations, the timing of data collection relative to the COVID-19 pandemic, country income classifications, geographic regions, and the specific vaccine categories under investigation.

To ensure statistical robustness and mitigate potential variability in subsequent analyses, specific childhood-related immunizations with a limited number of individual data points, including Bacillus Calmette-Guérin (BCG), Diphtheria, Pertussis, Tetanus (DPT), Hepatitis B vaccine (HBV), measles-containing vaccines, Pneumococcal conjugate vaccine (PCV), pertussis, polio, and rotavirus, were consolidated and combined into the “Childhood” category. This categorization allowed for a more reliable assessment of broader trends in pediatric vaccination acceptance.

#### 2.7.1. Meta-Analytic Model and Data Transformation

The primary assessment was the pooled prevalence of vaccine acceptance for the whole dataset. A random-effects meta-analysis was performed to account for anticipated between-study heterogeneity. To address the inherent constraints of proportional data and stabilize variance, a Logit transformation was applied using a Generalized Linear Mixed Model (GLMM) approach. Estimates were subsequently back-transformed to proportions for interpretability.

#### 2.7.2. Assessment of Heterogeneity

Statistical heterogeneity was quantified using the I^2^ statistic, representing the percentage of total variation across studies due to heterogeneity rather than chance, and the *τ*^2^ (tau-squared) statistic, representing the estimated amount of residual heterogeneity. Cochrane’s Q test was utilized to assess the significance of differences between defined subgroups. The methodological quality of all included studies was independently assessed using the Joanna Briggs Institute (JBI) Critical Appraisal Checklist for Studies Reporting Prevalence Data. And the publication bias was assessed using Egger’s linear regression test of funnel-plot asymmetry.

#### 2.7.3. Subgroup and Meta-Regression Analysis

To identify factors influencing vaccine acceptance, we conducted subgroup analyses and mixed-effects meta-regressions. Variables examined as moderators included different vaccine categories, economic status according to World Bank income classifications, geographic region according to WHO classification and phases of the COVID-19 pandemic.

#### 2.7.4. Temporal and Interaction Modeling

We first analyzed the temporal relationship among vaccines in a continuous style and categorical style (COVID-19). To improve model stability and interpretability of the intercept, the study midpoint was centered at the median study year (2018.55). Interaction models were constructed to evaluate whether temporal trends (slopes) and pandemic-related shifts varied significantly across vaccine category, income groups, and geographic regions. Specifically, triple-interaction terms (e.g., Vaccine Category × Year (centered)/COVID phases × Region/Income) were employed to test for divergent longitudinal trajectories.

#### 2.7.5. Visualization and Software

Data visualization was performed using ggplot2 (Version 3.5.1). Trend lines were generated using linear regression models (y ~ x), with point sizes scaled according to study variance (inverse-weighting). All statistical procedures were conducted in R (Version 4.4) using the meta and metafor packages. A two-sided *p*-value < 0.05 was considered statistically significant.

## 3. Results

### 3.1. Identified Literature

In total, 50,472 (PubMed-22,798; Scopus-15,717; Embase-11,810; Cochrane Library-147) records were identified from the databases using the combined searches (File S2: Database Keyword Search). All identified references were imported into a reference management system (Excel file), and duplicates (27,839) were removed prior to screening, resulting in 22,633 records. These records were then screened by title to remove clearly irrelevant records, resulting in 2815 articles retained. Titles and abstracts were independently screened against the eligibility criteria for the articles retained after title screening. Of these, 2381 records were excluded for not meeting eligibility criteria, yielding 434 articles eligible for full-text review. Full texts of the 434 articles were assessed in detail to determine final inclusion. Articles were excluded at this stage for reasons including ineligible population, vaccine category, study design or outcome measures. Articles categorized as uncertain were resolved through discussion and consensus among the review team. The final number of studies included in the systematic review is 264. The finalized studies were then further segregated for analysis into different data points if multiple vaccines or target populations were studied/evaluated in one study, resulting in 407 data points (File S3: Forest plot showing all the included datapoints in the systematic review).

### 3.2. Summary Descriptive Analyses

The descriptive characteristics of included vaccine acceptance studies in the systematic review are summarized in Table 1.

The majority of vaccine acceptance studies conducted between 2009 and 2024 (based on age group) focused on children aged 0–9 years (63.4%). More than half of the included studies were carried out in high-income countries, while only a small proportion (6.9%) originated from low-income countries. The human papillomavirus (HPV) vaccine was the most frequently studied, accounting for 39.3% of all studies. Approximately one-third (33.2%) of the studies (based on different vaccine categories) examined childhood vaccines, defined as those included in routine immunization programs.

### 3.3. General Trends in Vaccine Acceptance

#### 3.3.1. Overall Pooled Vaccine Acceptance

**Figure 3:**
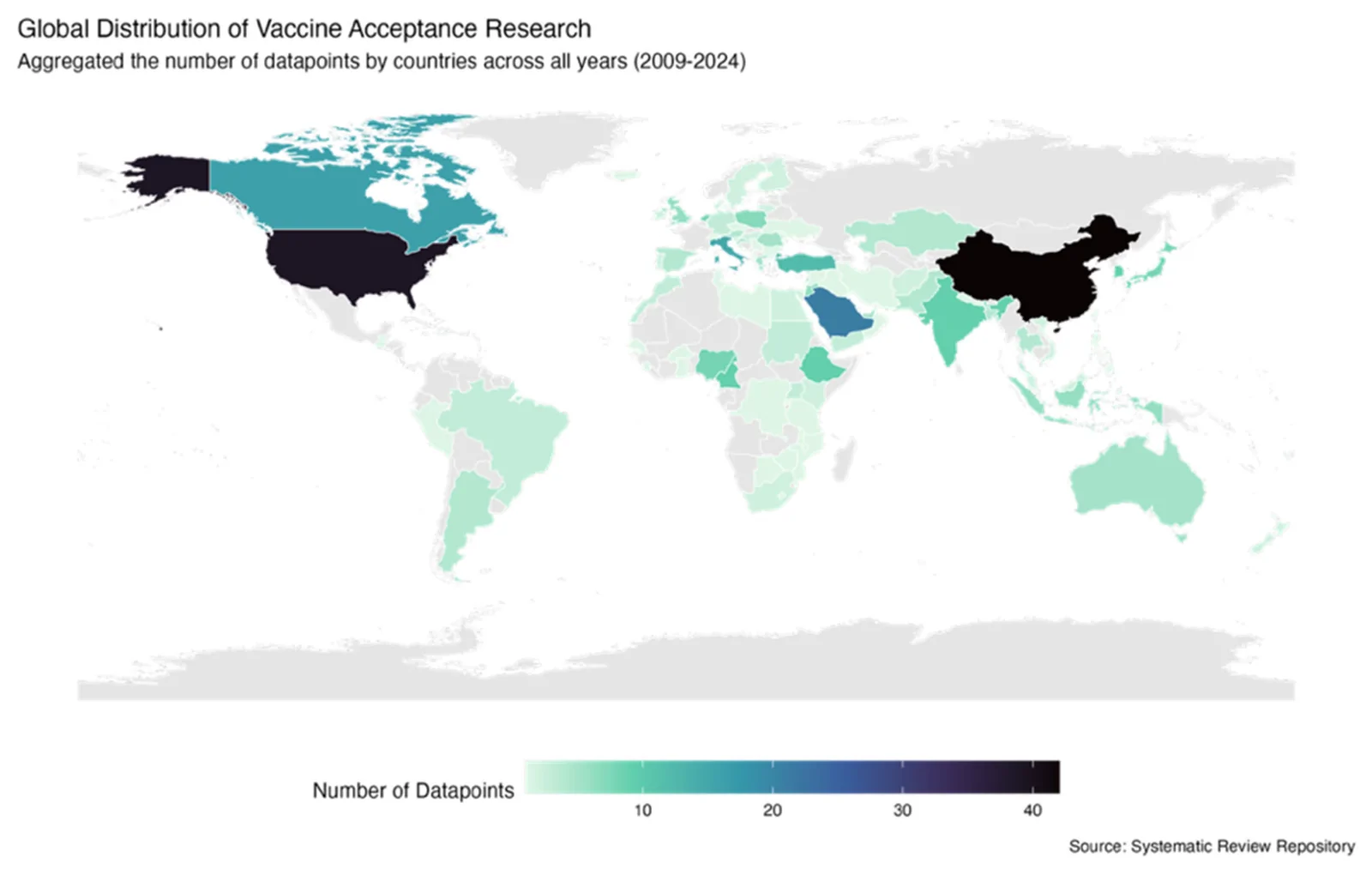
World map showing the global distribution of vaccine acceptance research (aggregate data points) during 2009–2024.

A total of 407 data points were included in the meta-analysis, which surveyed 650,772 individuals across 97 countries globally (Figure 3). Utilizing a random-effects model to account for anticipated inter-study heterogeneity, the overall pooled prevalence of vaccine acceptance across the entire study period (January 2009–December 2024) was **70.63%** (95% CI: 68.05–73.08%) (Table 2).

#### 3.3.2. Temporal Trends (2009–2024)

**Figure 4:**
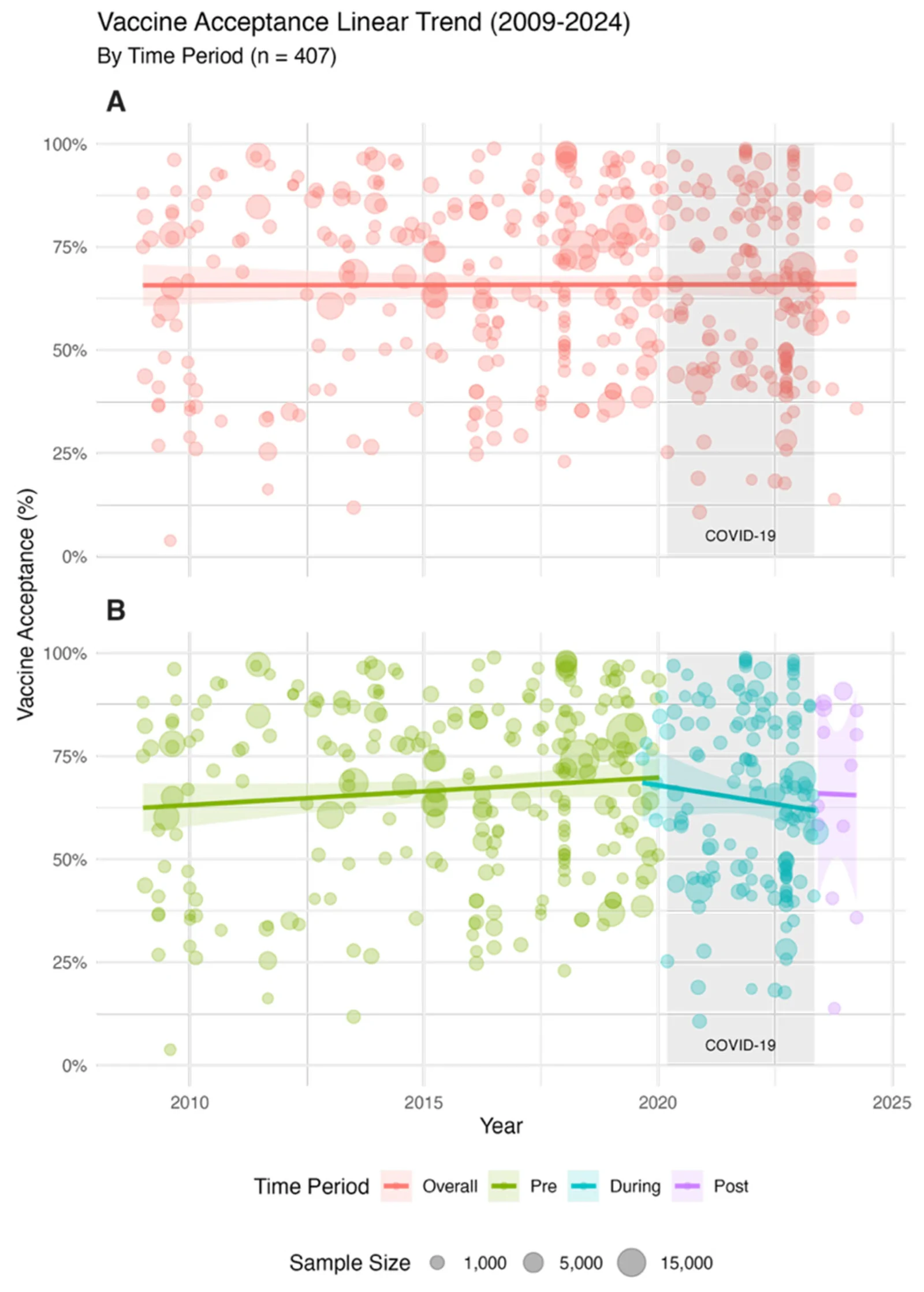
(**A**)—Overall vaccine acceptance trend from 2009 until 2024. (**B**)—Overall vaccine acceptance trend by time period relative to COVID-19.

To evaluate the continuous trend of vaccine acceptance over time, a meta-regression was performed using the midpoint year of data collection as a moderator. The results indicated no statistically significant linear trend in vaccine acceptance over the 15-year period (Estimate (β) = 0.0029, SE = 0.0147, *p* = 0.843), indicating a subtle, insignificant increase in the overall vaccine acceptance (from an estimated 70.0% to 70.3%) (Figure 4A). The model results suggest that vaccine acceptance remained relatively stable at the aggregate level, with the time variable failing to explain a significant portion of the observed variance (Q_M = 0.039, *p* = 0.843) (Table 3).

#### 3.3.3. Impact of the COVID-19 Pandemic Period

The pandemic’s influence was further examined by categorizing data into three distinct phases: pre-pandemic (baseline), during-pandemic, and post-pandemic. The omnibus test for moderators was non-significant (Q_M = 0.735, *p* = 0.693), indicating that the pandemic period did not exert a significant global effect on pooled vaccine acceptance rates (Figure 4B).

Compared to the Pre-pandemic baseline (Intercept logit estimate (β) = 0.919, *p* < 0.001) (predicted prevalence 70.9% [95% CI: 67.5% to 74.1%]), vaccine acceptance showed a slight decrease during the pandemic (Estimate (β) = −0.106, *p* = 0.417) (predicted prevalence: 69.3%) and a further slight reduction in the Post-pandemic period (Estimate (β) = −0.135, *p* = 0.703) (predicted prevalence: 68.7%). However, neither of these shifts reached statistical significance (Table 4).

#### 3.3.4. Heterogeneity Analysis

In all models, residual heterogeneity remained extremely high (I^2^ = 99.77%; tau^2^ = 1.535, *p* < 0.0001). This indicates that the vast majority of the variability in vaccine acceptance is not explained by time or the COVID-19 pandemic alone.

In addition, the results of the publication bias assessment using Egger’s linear regression test of funnel-plot asymmetry are presented in Supplementary Materials (File S4: Funnel-plot and linear regression test of asymmetry). Furthermore, all 264 included studies were critically appraised for methodological quality using the JBI critical appraisal checklist for studies reporting prevalence data. The appraisal demonstrated that methodological quality varied across the nine assessment domains. Detailed appraisal results are presented in Supplementary Materials (File S5: Critical Appraisal of Included Studies).

### 3.4. Vaccine-Specific Acceptance and Temporal Interactions

#### 3.4.1. Subgroup Meta-Analysis by Vaccine Category

A subgroup analysis was conducted to compare acceptance rates across different vaccine categories. Significant differences were observed between groups (Q_B = 93.77, df = 3, *p* < 0.0001). Childhood vaccines exhibited the highest pooled acceptance at 80.26% (95% CI: 76.90–83.23%, k = 169), followed by HPV vaccines at 66.48% (95% CI: 62.59–70.15%, k = 160). Influenza vaccines showed the lowest acceptance rate at 52.00% (95% CI: 47.47–56.50%, k = 73) (Figure 4). Due to a limited sample size (k = 5), the “General” vaccine category was excluded from subsequent regression analyses to ensure model stability (Table 1).

#### 3.4.2. Temporal Divergence: Interaction Between Time and Vaccine Category

To determine if temporal trends in vaccine acceptance differed by vaccine category, a multivariable meta-regression was performed with time centered at the study’s median year (year 2018.55). The inclusion of interaction terms between centered time and vaccine category revealed a significant moderation effect (Q_M = 86.37, df = 5, *p* < 0.0001).

For the reference group (Childhood vaccines), the model estimated a baseline logit acceptance of 1.44 (predicted prevalence: 80.9%) at the median year (*p* <0.0001) and a significant positive annual trend (β = 0.0739, *p* = 0.0032) (predicted prevalence from 2009: 67.4% to 2024: 86.1%), indicating that acceptance for routine childhood immunizations improved over the study period.

**Figure 5:**
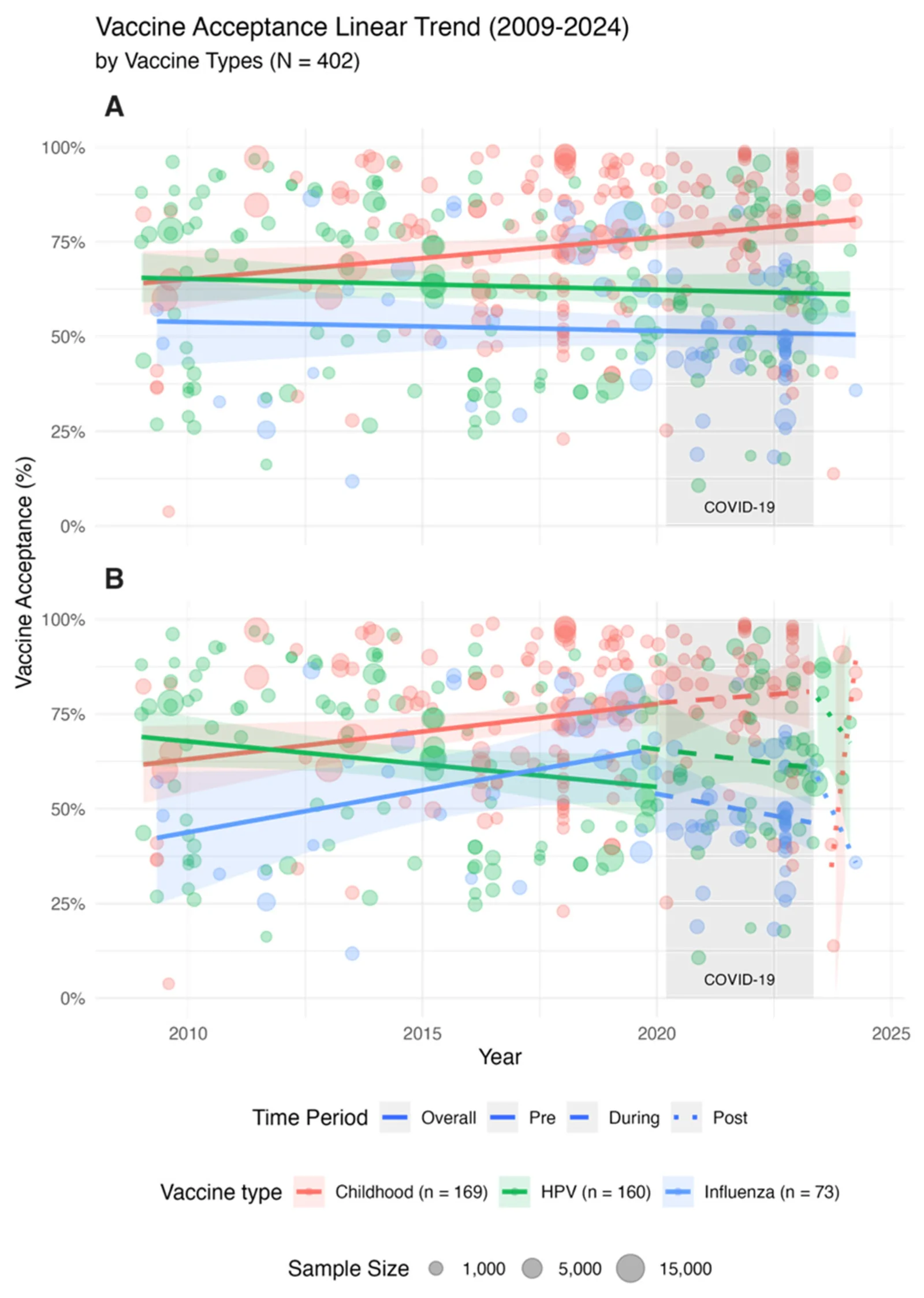
(**A**)—Vaccine acceptance trend by vaccine type (category). (**B**)—Vaccine acceptance trend by vaccine types (categories) and time period.

However, significant negative interaction terms were observed for both HPV (β = −0.0947, *p* = 0.0026) (predicted prevalence from 2009, 69.9% to 2024, 61.2%) and Influenza vaccines (β = −0.0855, *p* = 0.0457) (predicted prevalence from 2009, 55.1% to 2024, 50.7%). These interactions indicate that the temporal trajectories for these vaccines diverged significantly from the upward trend seen in childhood vaccines. Specifically, while childhood vaccine acceptance increased, the net annual trends for HPV and Influenza vaccines were negative (calculated as β_net = −0.0208 and β_net = −0.0116, respectively), associated with a gradual erosion of acceptance for these specific vaccines throughout the 2009–2024 period. Additionally, at the median year of the study, both HPV (β = −0.795, *p* = <0.0001) (predicted prevalence: 64.4%) and Influenza vaccines (β = −1.350, *p* = <0.0001) (predicted prevalence: 52.2%) had significantly lower baseline acceptance levels compared to childhood vaccines (Figure 5A) (Table 5).

#### 3.4.3. Pandemic Impact Across Vaccine Categories

The interaction between vaccine category and the COVID-19 pandemic phases was further examined (Q_M = 83.89, *p* < 0.0001). The analysis revealed that the pandemic may differ in vaccine acceptance by vaccine type:

- *Childhood Vaccines:* Showed a significant increase in acceptance during the COVID-19 period compared to the pre-pandemic baseline (estimate (β) = 0.5144, *p* = 0.0119). The predicted prevalence during the COVID-19 period for childhood vaccines is 85.7%, compared to the predicted prevalence in the pre-COVID period: 78.2%.
- *HPV Vaccines:* Compared with the average acceptance of childhood vaccines during the COVID-19 period, the HPV vaccine acceptance remained almost entirely flat (estimate (β) = −0.4970, *p* = 0.0861), with its predicted prevalence as 65.3% and predicted pre-COVID prevalence as 64.9%.
- *Influenza Vaccines:* Exhibited a strong negative interaction during the pandemic (estimate (β) = −0.8281, *p* = 0.0195), dropping predicted prevalence to 49.2% compared to the pre-COVID: 57.0%. The magnitude of this negative interaction suggests that the pandemic is associated with a net decrease in willingness to receive influenza vaccinations, diverging sharply from the trend seen in routine childhood shots.

In the post-pandemic period, no significant shifts were detected for any vaccine category relative to their pre-pandemic levels (*p* > 0.10), though the negative baseline for HPV and Influenza persisted (Figure 5B) (Table 6).

Please note that general vaccines are excluded from the analysis because there were very few data points (only 5) associated with this vaccine category.

### 3.5. Role of Economic Status in Vaccine Acceptance

#### 3.5.1. Subgroup Meta-Analysis by Income Level

The dataset was stratified by World Bank income classifications to assess the impact of economic development on vaccine willingness (k = 400). Significant differences were found between income groups (Q_B = 14.42, df = 3, *p* = 0.0024). Lower-middle-income countries (LMICs) reported the highest pooled vaccine acceptance at 80.28% (95% CI: 74.52–84.99%), followed by Low-income countries at 75.08% (95% CI: 65.47–82.72%). In contrast, acceptance rates were notably lower in Upper-middle-income countries (68.78%, 95% CI: 62.93–74.09%) and reached their lowest point in High-income countries (67.49%, 95% CI: 64.00–70.79%).

#### 3.5.2. Triple Interaction: Vaccine Category, Economic Status, and Continuous Time

To evaluate whether the longitudinal trajectories of specific vaccines were moderated by the economic status of the study country, a triple-interaction mixed-effects meta-regression was performed (k = 400). Time was centered at the median year of 2018.552 to provide a meaningful intercept and reduce multicollinearity. The omnibus test of moderators was highly significant (Q_M= 125.01, df = 23, *p* = <0.0001), indicating that the combination of vaccine category, income level, and time significantly explained variations in vaccine acceptance.

The reference group—Childhood vaccines in Low-income countries at the median year—demonstrated a high estimated logit prevalence of 1.933 (<0.001). At this baseline, HPV vaccine acceptance was significantly lower than childhood vaccines (β = −1.14, *p* = 0.0377), while acceptance in Upper-middle-income countries was marginally lower than in low-income settings (β = −0.877, *p* = 0.0528).

Despite the overall significance of the model, none of the triple-interaction coefficients reached statistical significance (all *p* > 0.40). Similarly, the two-way interactions between time and income level, and time and vaccine category within this multivariable context, were non-significant.

These results suggest that while baseline levels of vaccine acceptance differ significantly depending on the vaccine category and the country’s income group, the annual rate of change (the temporal slope) for Childhood, HPV, and Influenza vaccines did not vary significantly across different economic strata. In other words, the divergent temporal trends observed between routine childhood immunizations and “discretionary” vaccines (HPV and Influenza) appear to be a global phenomenon that persists regardless of a country’s specific income level.

#### 3.5.3. Triple Interaction: Vaccine Category, Economic Status, and COVID-19 Period

**Figure 6:**
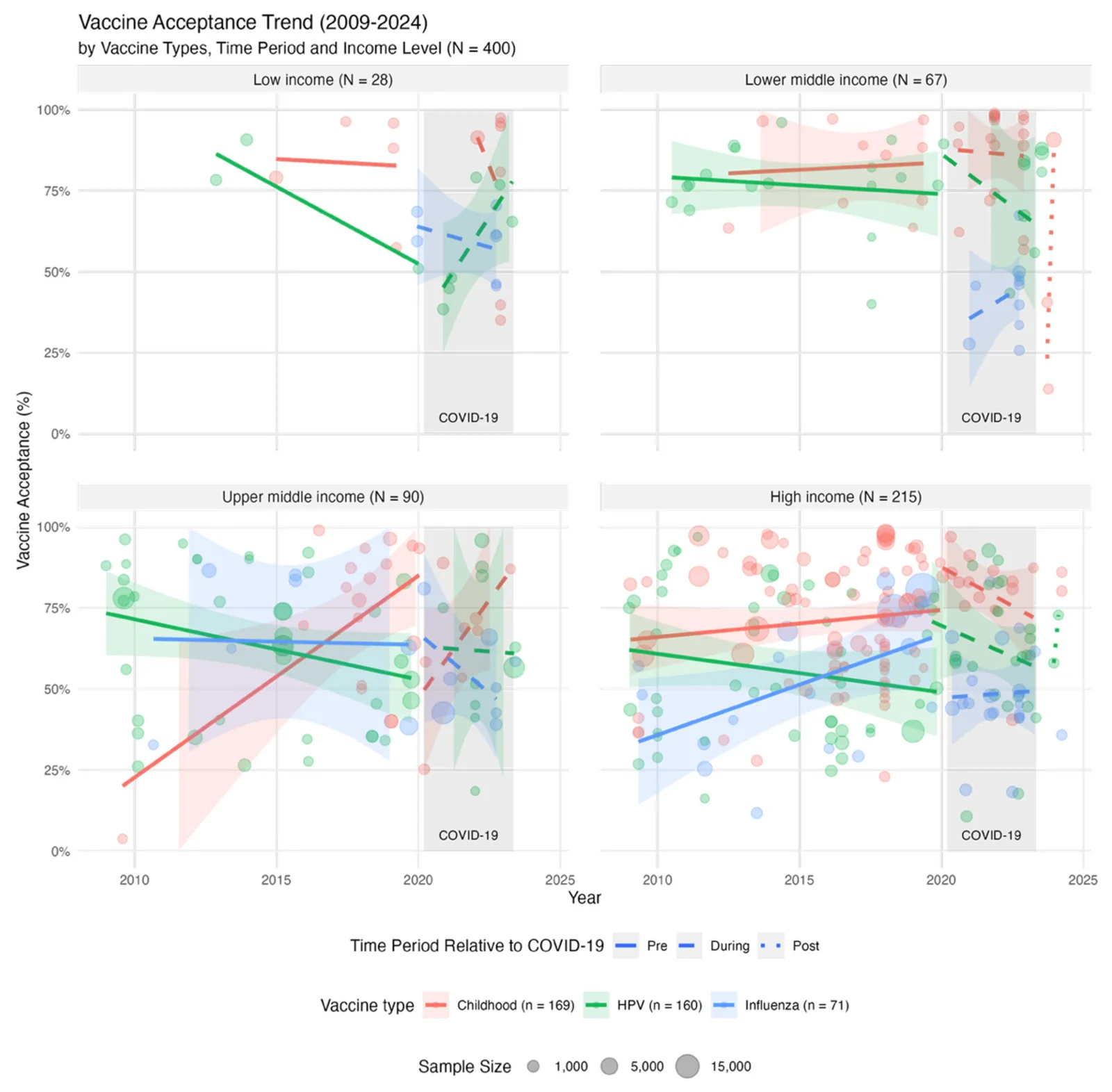
Vaccine acceptance trend by vaccine types (categories) and time period relative to COVID-19 pandemic across different income levels.

A more granular analysis was conducted to see how the pandemic shifted acceptance for specific vaccines across different income levels (Q_M = 135.88, *p* < 0.0001). Several key findings emerged (Figure 6):

- *Baseline Disparities:* High-income countries showed a marginally lower baseline acceptance compared to lower-income counterparts at the start of the study period (estimate (β) = −0.82, *p* = 0.099).
- *The Post-Pandemic Shift in LMICs:* A significant negative effect was observed in the post-pandemic period specifically for LMICs (estimate (β) = −2.32, *p* = 0.0268). While these countries started with high baseline acceptance, they experienced a decrease in vaccine acceptance following the end of the COVID-19 emergency.
- *HPV Vaccine Resilience in LMICs:* Interestingly, for the HPV vaccine in LMICs during the post-pandemic phase, there was a positive interaction trend (estimate (β) = 2.45, *p* = 0.0954). This suggests that despite a general decline in broad vaccine acceptance in these regions post-COVID, HPV-specific acceptance showed signs of comparative resilience or recovery.

### 3.6. Geographic Variations in Vaccine Acceptance

#### 3.6.1. Subgroup Meta-Analysis by WHO Region

A subgroup analysis was conducted to compare pooled vaccine acceptance across geographic boundaries (k = 401). There was significant geographic heterogeneity (Q_B = 49.18, df = 5, *p* < 0.0001).

The African Region demonstrated the highest level of vaccine acceptance at 85.59% (95% CI: 79.89–89.87%), closely followed by the South-East Asia Region at 81.12% (95% CI: 75.59–85.64%). The Region of the Americas followed with an acceptance rate of 74.72% (95% CI: 68.17–80.30%). Conversely, the lowest acceptance rates were observed in the Western Pacific Region (62.96%, 95% CI: 57.10–68.46%), the Eastern Mediterranean Region (63.15%, 95% CI: 57.14–68.78%), and the European Region (67.80%, 95% CI: 62.85–72.38%).

#### 3.6.2. Triple Interaction: Vaccine Category, Region and Continuous Time

To evaluate the extent to which geographic region moderated the temporal trajectories of specific vaccine categories, a triple-interaction mixed-effects meta-regression was conducted (k = 401). Centering the time variable at the study’s median year (2018.552) provided a stable baseline for comparing regional and vaccine-specific intercepts. The omnibus test for moderators was highly significant (Q_M = 166.74, df = 32, *p* = <0.0001), indicating that the model effectively captured variations in vaccine acceptance across regions and categories.

The reference group—Childhood vaccines in the African Region at the median year—exhibited a high estimated logit prevalence of 2.678 (*p* < 0.0001). At this midpoint, significant regional disparities were evident for childhood vaccines; compared to Africa, baseline acceptance was significantly lower in the European Region (β = −1.603, *p* < 0.0001), the Western Pacific Region (β = −1.557, *p* = 0.0008), the South-East Asia Region (β = −1.170, *p* = 0.0285), and the Eastern Mediterranean Region (β = −0.924, *p* = 0.0423).

Regarding vaccine-specific differences at the median year, HPV vaccine acceptance in Africa was significantly lower than childhood vaccines (β = −1.594, *p* = 0.0003). However, significant two-way interactions between vaccine category and region were observed: the gap between HPV and childhood vaccines was significantly smaller in the South-East Asia Region (β = 1.516, *p* = 0.0226) and the European Region (β = 1.007, *p* = 0.0490) compared to the gap observed in Africa.

The analysis of temporal trends showed that the annual slope for childhood vaccines in the African Region was not statistically significant (β = −0.114, *p* = 0.2735). Critically, all triple-interaction coefficients failed to reach statistical significance (all *p* > 0.26). This suggests that while the baseline levels of acceptance for different vaccines vary greatly by region, the rate of change over time for Childhood, HPV, and Influenza vaccines was relatively consistent across all geographic regions. The divergence in trajectories between these vaccine categories appears to be a global trend that is not significantly altered by regional geographic context.

#### 3.6.3. Triple Interaction: Vaccine Category, Region, and COVID-19 Period

**Figure 7:**
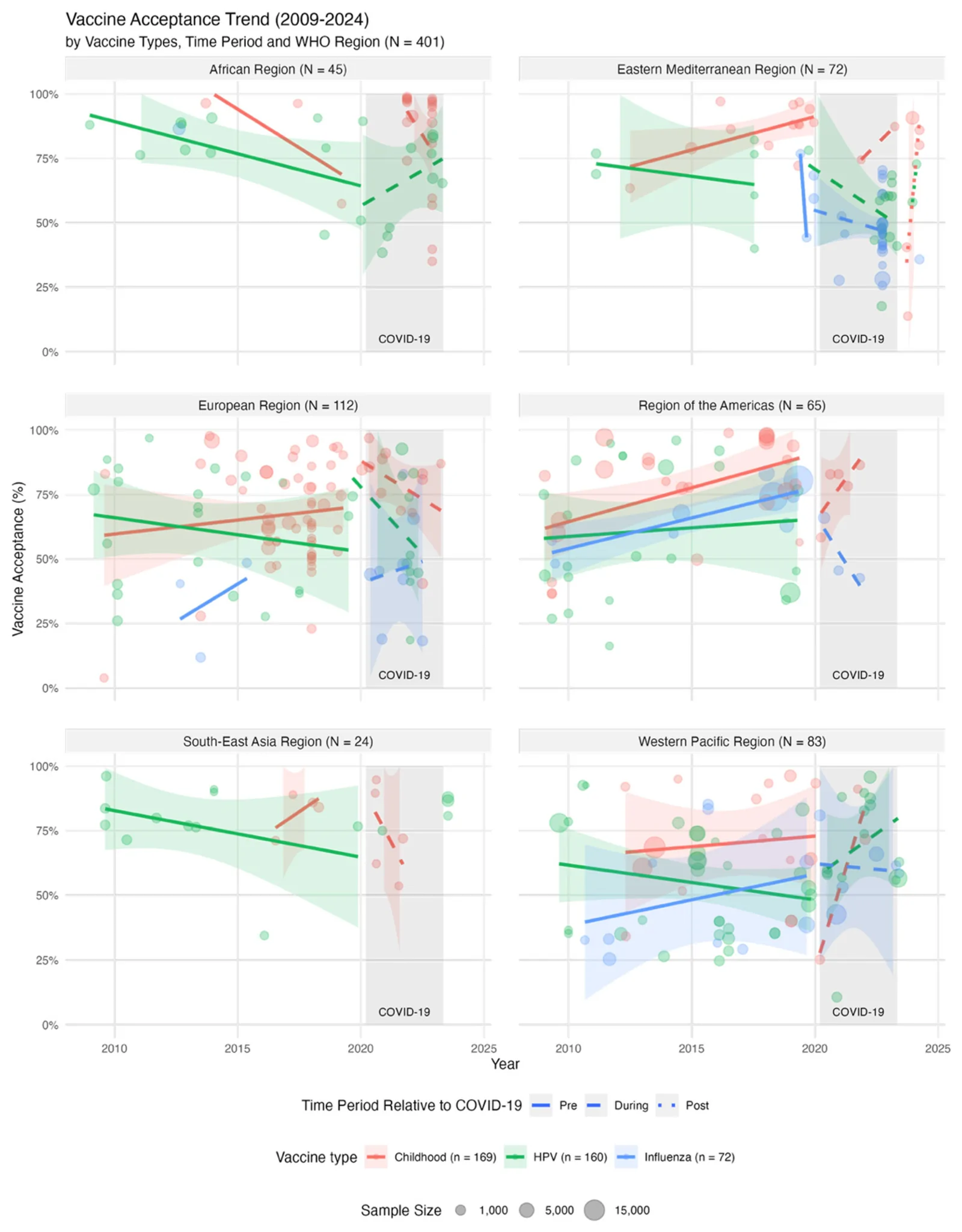
Vaccine acceptance trend by vaccine types (categories) and time period relative to COVID-19 pandemic across different WHO regions.

The impact of the pandemic on vaccine categories across different regions was analyzed using a multi-level interaction model (Q_M = 170.08, *p* < 0.0001) (Figure 7).

- Pre-Pandemic Baselines: The analysis confirmed a highly significant baseline for routine childhood vaccines in the African Region (Intercept = 2.26, *p* = 0.0002). In contrast, the European Region had a significantly lower pre-pandemic baseline for childhood vaccines compared to Africa (estimate (β) = −1.38, *p* = 0.0277).
- Regional Resilience during COVID-19: No significant two-way or three-way interactions reached the standard *p* < 0.05 threshold for the pandemic period. However, there was a marginal positive interaction for HPV vaccines in the Western Pacific Region during the pandemic (estimate (β) = 1.83, *p* = 0.0923), suggesting a potential resilience or targeted effort for HPV vaccination in that region despite global pandemic pressures.
- Post-Pandemic Stability: Regional vaccine sentiments did not show significant new shifts in the post-pandemic period relative to their own pre-pandemic baselines, indicating that the geographic disparities observed before 2020 have largely persisted into the post-emergency era.

## 4. Discussion

Our analysis demonstrated that most vaccine acceptance studies conducted between 2009 and 2024 originated from high-income countries, with limited representation from low-income countries. This likely reflects structural constraints rather than lack of relevance, including limited research infrastructure, funding, and trained personnel, weak data systems, difficulties reaching rural populations and competing health priorities [22,23]. Additionally, concentration of research on vaccine acceptance in a few high- and upper-middle-income countries may further contribute to the underrepresentation of LICs in the studies [24,25]. Future efforts should prioritize strengthening local research capacity, supporting collaborative and locally led studies, and developing standardized yet culturally adaptable approaches for measuring vaccine acceptance in low-income settings. Improved representation of low-income settings will be essential for designing equitable and context-specific immunization strategies.

The overall pooled vaccine acceptance in our study was 70.63%, based on 650,772 individuals across 97 countries, and remained relatively stable during the 2009–2024 time period. This is comparable to findings by Wang et al., who reported vaccine confidence of 77% from a total of 122,146 individuals across 141 countries between 2015 and 2022 [26]. However, Wiegand et al. reported declining vaccine confidence between 2015 and 2022 (surveyed 165,729 individuals across 55 countries) using measures focused on vaccine importance, safety and effectiveness of vaccines [27]. The discrepancy in findings may be partly explained by differences in the measurement of vaccine acceptance. Specifically, the referenced study assessed vaccine confidence using a validated three-item battery measuring perceptions of vaccine importance, safety, and effectiveness on a four-point Likert scale, whereas our study measured overall vaccine acceptance as a percentage, capturing a broader construct. In addition, another study by the Vaccine Confidence Project examined global trends in vaccine confidence between September 2015 and December 2019, across 149 countries (including 284,381 individuals) and demonstrated that vaccine confidence varied considerably across countries and over time, with some regions showing increasing confidence while others reported declines or fluctuating trends [11].

Although a slight decrease in vaccine acceptance was observed during and after the COVID-19 pandemic, these changes were modest and likely driven by contextual factors, including levels of trust in healthcare providers, government and public health institutions, exposure to vaccine-related information and misinformation, perceived disease risk, healthcare access, previous experiences with vaccination, sociopolitical environment, etc., rather than time alone. Previous evidence suggests that vaccine acceptance is generally stable, with variations more strongly associated with vaccine category, geographic region, economic setting, trust in health systems, and perceptions of vaccine safety and effectiveness [24,25,28]. Individual-level factors, including education, income, prior vaccination behavior, and trust in health authorities, also play an important role in shaping vaccine acceptance across settings [29,30].

The observed gradient in vaccine acceptance-highest for childhood vaccines and lower for HPV and influenza vaccines-is consistent with vaccine-specific determinants reported in previous studies. Childhood vaccines showed consistently higher and more stable acceptance, likely reflecting their long-standing inclusion in routine immunization programs and stronger social acceptance [29]. In contrast, HPV vaccine acceptance showed a declining trend since 2009, including during and after the COVID-19 pandemic, potentially influenced by safety concerns, sociocultural beliefs, and trust in health systems [31,32]. Influenza vaccine acceptance increased prior to the COVID-19 pandemic, possibly reflecting the impact of the 2009 H1N1 pandemic on public risk perception and vaccination behavior, but declined during the COVID-19 period, which may relate to pandemic-related vaccine fatigue, misinformation and increasing polarization around vaccination [11].

The findings also suggest an inverse relationship between country income level and vaccine acceptance, with greater vaccine hesitancy often observed in high-income countries, consistent with findings by Wang et al., who also noted the highest proportion of vaccine confidence in LICs and the lowest in the HICs [26]. Although trends remained relatively stable across income groups over time, the COVID-19 pandemic appeared to disrupt acceptance patterns, particularly in LMICs, where post-pandemic declines may reflect health system strain and the impact of misinformation [25]. In addition, the emergence of influenza vaccine acceptance studies in LICs and LMICs during the pandemic—despite no studies prior to COVID-19—likely reflects increased research prioritization driven by heightened global attention to respiratory infections and vaccination behaviors. This is consistent with evidence that influenza vaccination has historically received limited focus in these settings due to constrained resources and competing health priorities [33].

The analysis across WHO regions demonstrated marked geographic differences in vaccine acceptance, with higher acceptance in the African and South-East Asian regions and lower levels in the Western Pacific and European regions. This is consistent with findings by Wang et al. showing the highest vaccine confidence in SEAR and the lowest in Europe [26]. The persistently lower baseline vaccine acceptance in Europe prior to the COVID-19 pandemic reflects long-standing vaccine confidence challenges rather than a pandemic-driven effect. Pre-COVID global analyses already identified lower confidence in vaccine safety, importance, and effectiveness in several European countries compared with other regions [11,34]. Within-country evidence further highlights persistent hesitancy clusters in some countries driven by safety concerns, distrust in health systems, and variable confidence in health authorities [35]. Higher acceptance in African and South-East Asian settings may reflect stronger perceived disease risk, reliance on routine immunization programs, and greater trust in vaccination as a public health measure [25,29]. Together, these findings suggest that regional differences are driven by underlying sociocultural, epidemiological, and informational contexts rather than geographic location alone.

The relatively balanced distribution of HPV vaccine acceptance studies across all WHO regions and income levels likely reflects the global prioritization of HPV vaccination within cervical cancer prevention strategies. International initiatives led by WHO and Gavi, the Vaccine Alliance have supported demonstration projects, feasibility assessments, and country-level evaluations of HPV vaccine acceptability before and during implementation, contributing to a more geographically balanced evidence base [36,37]. Interestingly, HPV vaccine acceptance in LMICs showed a positive post-pandemic interaction trend despite an overall decline in vaccine acceptance across these settings. Although this trend did not reach statistical significance, it may suggest that HPV vaccine acceptance has been comparatively more resilient than that of other vaccines. This resilience may be attributable to sustained investments in HPV vaccine introduction through phased implementation, school-based delivery platforms, and community engagement, which have remained central to cervical cancer prevention programs in many LMICs [36,37]. Such sustained implementation efforts may have helped maintain public confidence despite broader disruptions to immunization services during the COVID-19 pandemic. Furthermore, unlike seasonal influenza vaccination, HPV vaccination targets a defined adolescent population and is delivered through organized programs rather than repeated annual vaccination, potentially making acceptance less susceptible to pandemic-related vaccine fatigue. Nevertheless, HPV vaccine acceptance remained lower than that of routine childhood vaccines, indicating that concerns regarding vaccine safety, limited awareness, and sociocultural beliefs continue to influence uptake [31,32]. These findings suggest that sustained investment in structured vaccine introduction and community engagement may help strengthen vaccine acceptance during future public health disruptions.

Although validated tools such as the WHO SAGE Vaccine Hesitancy Scale (VHS) and Parent Attitudes about Childhood Vaccines (PACV) have strengthened vaccine attitude research, differences in scoring systems and interpretation limit comparability across studies. Vaccine hesitancy is a multidimensional and context-specific construct, and existing tools primarily assess attitudes rather than direct acceptance, leading to variability in reporting [38,39]. Moreover, systematic reviews have identified substantial heterogeneity in available instruments, with no single validated tool capable of consistently assessing vaccine hesitancy across different vaccines, populations, and settings, limiting cross-study comparability [40,41]. The conversion of scale-based outputs (e.g., low–high hesitancy categories) into percentage-based acceptance estimates further risks misclassification and oversimplification, as these scales are not designed to directly quantify uptake. These limitations highlight the need for a harmonized, globally applicable, and easy-to-interpret tool that can integrate both behavioral intent and measurable vaccine acceptance trends.

Vaccine acceptance is not a homogeneous phenomenon and may vary according to sociocultural context, regional events, public health policies and experiences with vaccination programs [2]. The included studies were conducted across diverse geographical settings with differences in vaccine introduction timelines, immunization strategies and approaches used to assess vaccine acceptance [11]. Moreover, most studies were not specifically designed to evaluate vaccine acceptance prevalence. Variations in measurement tools, study designs, and outcome definitions likely contributed to the observed heterogeneity [42], while some context-specific factors influencing acceptance may not have been captured. Nevertheless, this review aimed to assess global patterns and trends in routine vaccine acceptance from 2009 to 2024 rather than estimate a single universal acceptance level. Subgroup and meta-regression analyses were therefore performed to explore potential sources of variation across vaccine categories, geographic locations, economic settings and COVID-19 pandemic periods.

This systematic review has some limitations that should be considered when interpreting the findings. First, substantial heterogeneity across studies, including differences in populations, vaccine categories, study designs, and settings, may limit comparability and affect pooled estimates. Additionally, variations in the definitions and measurement of vaccine acceptance across studies may have also introduced heterogeneity and limited direct comparability of pooled estimates. Residual dependence among multiple estimates from the same study cannot be completely excluded and may affect the precision of pooled estimates. Second, geographic and economic representation was uneven, with underrepresentation of low-income countries and the African region, which may limit generalizability. Importantly, within-region heterogeneity and country-level variation may not be fully captured, and regional estimates may mask substantial differences between countries. In addition, evidence from low-income settings may be subject to selection bias, as published studies are more likely to originate from countries with stronger research capacity, better immunization infrastructure, or ongoing vaccine-related programs, potentially skewing representativeness. Moreover, heterogeneity in study designs, sampling approaches and vaccine acceptance measurement tools may have introduced potential bias and limited comparability across studies. Third, inclusion was restricted to studies reporting percentage-based vaccine acceptance outcomes, potentially excluding studies using validated scale-based measures such as the WHO SAGE VHS and PACV. Fourth, publication and language bias cannot be excluded as studies from certain regions may be less likely to be indexed or published. Fifth, temporal comparisons across pre-, during-, and post-pandemic periods should be interpreted cautiously, as observed differences may reflect contextual influences rather than true temporal trends. The limited number of post-COVID data points may also have restricted the ability to fully capture temporal variations in vaccine acceptance. Sixth, although PubMed and Cochrane Library provide a substantial evidence base, the restriction of databases may have resulted in the omission of potentially eligible studies indexed in other databases. Seventh, the exclusion of pandemic-specific vaccines, including COVID-19 vaccines, limits assessment of attitudes toward emergency vaccination programs and their potential influence on broader vaccine acceptance. Finally, the exclusion of studies focusing on high-risk populations, such as older adults and individuals with comorbidities, limits the generalizability of the influenza vaccine acceptance estimates to primary target populations for seasonal influenza vaccination.

## 5. Conclusions

This review highlights that vaccine acceptance remained relatively stable between 2009 and 2024, but showed substantial variation across vaccine categories, regions, and contexts rather than uniform temporal trends. Clear vaccine-specific differences were evident, with consistently higher acceptance for childhood vaccines and lower, more variable acceptance for HPV and influenza vaccines, indicating that vaccine acceptance is strongly shaped by vaccine-specific determinants in addition to broader contextual factors. The reasons underlying these differences, particularly for HPV vaccination, are not yet fully understood and warrant further investigation, as sociocultural influences, risk perception, and implementation strategies may interact differently across settings. Despite overall stability, important regional and economic disparities persist, alongside underrepresentation of certain settings, highlighting the need to strengthen evidence generation and incorporate additional data sources for more balanced global estimates. Furthermore, inconsistencies in measurement approaches across studies underscore the need for a harmonized, globally applicable, and easy-to-interpret tool to improve comparability and better integrate behavioral intent with observed acceptance patterns.

## Figures and Tables

**Table 1:** Characteristics of included vaccine acceptance studies.

Characteristics	n (%)
Sample Size: N = 407	
Summary	Med: 591 (IQR: 746, Range: 27–39,617)
**Vaccine Target group**	
Adolescent	91 (22.4%)
Adult	54 (13.3%)
Children	258 (63.4%)
Elderly	4 (1%)
**COVID-19 pandemic**	
Before	251 (61.7%)
During	143 (35.1%)
After	13 (3.2%)
**Country Income group (World Bank Category)**	
Low-income	28 (6.9%)
Lower-middle-income	67 (16.5%)
Upper-middle-income	91 (22.5%)
High-income	219 (54.1%)
*Missing*	2
**Country Region (WHO Category)**	
African Region (AFR)	45 (11.1%)
Eastern Mediterranean Region (EMR)	72 (17.7%)
European Region (EUR)	114 (28.1%)
Region of the Americas (AMR)	66 (16.3%)
South-East Asia Region (SEAR)	24 (5.9%)
Western Pacific Region (WPR)	85 (20.9%)
*Missing*	1
**Studied vaccines**	
BCG	1 (0.2%)
Childhood	135 (33.2%)
DPT	5 (1.2%)
General *	5 (1.2%)
HBV	1 (0.2%)
HPV	160 (39.3%)
Influenza **	73 (17.9%)
Measles	11 (2.7%)
PCV	5 (1.2%)
Pertussis	1 (0.2%)
Polio	2 (0.5%)
Rota	8 (2%)

* General vaccine refers to the vaccines that are not specified in the literature on the target group and the specific vaccine category. ** Influenza vaccine includes the following age groups: children, adolescents and adults, including the elderly.

**Table 2:** Meta-analytic results for overall vaccine acceptance w.r.t. vaccine categories.

Outcome/Subgroup	K (Studies)	Proportion	95% CI	*τ* ^2^	τ
Overall vaccine acceptance	407	0.7063	0.6805–0.7308 ***	1.5383	1.2403
Childhood vaccine	169	0.8026	0.7690–0.8323	1.7318	1.3160
HPV vaccines	160	0.6648	0.6259–0.7015	1.1837	1.0880
Influenza vaccines	73	0.5200	0.4747–0.5650	0.6178	0.7860
General vaccines	5	0.7416	0.6115–0.8395	0.4626	0.6802

*p*-value: <0.0001—***.

**Table 3:** Multivariable meta-regression model predicting the pooled estimate of vaccine acceptance through the continuous time period (2009–2024).

Predictor/Variable	Estimate (β)	SE	z-Value	*p*-Value	95% CI
Time of Studies from 2009 until 2024	−4.9898	29.5860	−0.1687	0.8661	−62.9773–52.9976
Study Time Mid-Year (average between 2009 and 2024)	0.0029	0.0147	0.1983	0.8428	−0.0258–0.0316

Estimate (β)—regression coefficient; SE—Standard error; 95% CI—95% Confidence Interval.

**Table 4:** Multivariable meta-regression model predicting the pooled estimate of vaccine acceptance with reference to the COVID-19 pandemic.

Predictor/Variable	Estimate (β)	SE	z-Value	*p*-Value	95% CI
Pre-COVID-19 (January 2009–February 2020)	0.9190	0.0787	11.6814	<0.0001	0.7648–1.0732 ***
During COVID-19 (Mar 2020–May 2023)	−0.1060	0.1305	−0.8118	0.4169	−0.3618–0.1499
Post-COVID-19 (June 2023–December 2024)	−0.1350	0.3536	−0.3819	0.7025	−0.8281–0.5580

Estimate (β)—regression coefficient; SE—Standard error; 95% CI—95% Confidence Interval. *p*-value: <0.0001—***.

**Table 5:** Multivariable meta-regression model predicting the pooled estimate of vaccine acceptance: Interaction between continuous time (years) and vaccine categories.

Predictor/Variable	Estimate (β)	SE	z-Value	*p*-Value	95% CI
Baseline (Childhood vaccines)	1.4400	0.0887	16.2427	<0.0001	1.2662–1.6137 ***
Annual Trend Based on Childhood Vaccines	0.0739	0.0251	2.9491	0.0032	0.0248–0.1231 **
HPV vaccines (Baseline difference)	−0.7951	0.1314	−6.0491	<0.0001	−1.0527–−0.5375 ***
Influenza vaccines (Baseline difference)	−1.3498	0.1628	−8.2892	<0.0001	−1.6690–−1.0307 ***
Interaction: HPV vaccines with Year	−0.0947	0.0314	−3.0140	0.0026	−0.1564–−0.0331 **
Interaction: Influenza vaccines with Year	−0.0855	0.0428	−1.9981	0.0457	−0.1694–−0.0016 *

Estimate (β)—regression coefficient; SE—Standard error; 95% CI—95% Confidence Interval. *p*-value: <0.0001—***; <0.001—**; <0.01—*.

**Table 6:** Multivariable meta-regression model predicting the pooled estimate of vaccine acceptance: Interaction between specific COVID-19 timepoint and vaccine categories.

Predictor/Variable	Estimate (β)	SE	z-Value	*p*-Value	95% CI
Childhood vaccines and pre-COVID-19	1.2761	0.1091	11.6947	<0.0001	1.0622–1.4900 ***
Childhood vaccines and during COVID-19	0.5144	0.2045	2.5154	0.0119	0.1136–0.9153 *
Childhood vaccines and post-COVID-19	−0.6162	0.5378	−1.1458	0.2519	−1.6702–0.4378
HPV vaccines and pre-COVID-19	−0.6613	0.1584	−4.1757	<0.0001	−0.9717–−0.3509 ***
HPV vaccines and during COVID-19	−0.4970	0.2896	−1.7162	0.0861	−1.0647– 0.0706
HPV vaccines and post-COVID-19	1.1907	0.7301	1.6310	0.1029	−0.2402–2.6216
Influenza vaccines and pre-COVID-19	−0.9928	0.2550	−3.8934	<0.0001	−1.4926–−0.4930 ***
Influenza vaccines and during COVID-19	−0.8281	0.3545	−2.3356	0.0195	−1.5229–−0.1332 *
Influenza vaccines and post-COVID-19	0.2122	1.0165	0.2087	0.8347	−1.7802–2.2045

Estimate (β)—regression coefficient; SE—Standard error; 95% CI—95% Confidence Interval. *p*-value: <0.0001—***; <0.01—*.

## Data Availability

The data used in this systematic review are derived from publicly available published studies and are included within the manuscript and its references. The R scripts used for data analysis are not publicly available due to privacy and internal research considerations but may be made available from the corresponding author upon reasonable request.

## Supplementary Materials

The following supporting information can be downloaded at https://www.mdpi.com/article/10.3390/vaccines14080663/s1, File S1: PRISMA checklist; File S2: Database Keyword Search; File S3: Forest plot showing all the included data points in the systematic review; File S4: Funnel-plot and linear regression test of asymmetry; File S5: Critical Appraisal of Included Studies.
