# Supplementary material for: Mapping the Global Landscape of Vaccine Acceptance Among the General Population from 2009 to 2024: A Systematic Review Across COVID-19 Pandemic Phases, Vaccine Categories, and Economic Strata
Source: Vaccines (Basel). 2026 Jul 29;14(8):663. doi: 10.3390/vaccines14080663 (PMC13517538; doi:10.3390/vaccines14080663)
Supplement: Supplementary file 1 [file vaccines-14-00663-s001.zip › File S2 - Database Keyword Search.pdf]

**File S2: Database Keyword Search**

**Database keyword search**

((Vaccin*) OR (Immuni*)) AND ((Hesitan*) OR (Accept*) OR (Uptake) OR (refus*) OR (confidence) OR (trust) OR (perception) OR (anti-vacci*) OR (reject*) OR (delay) OR (intent) OR (misconception)) AND ((child*) OR (general) OR (influenza)) NOT ((AZD1222) OR (ChAdOx1 nCoV-19) OR (ChAdOx1-S) OR (AZD2816) OR (Vaxzevria) OR (Covishield) OR (Comirnaty) OR (BNT162b2) OR ("mRNA vaccine") OR (Jcovden) OR ("Ad26.COV2.S") OR (JNJ-78436735) OR (Ad26COVS1) OR (VAC31518) OR (Spikevax) OR (mRNA-1273) OR (CX-024414) OR (Moderna) OR (TAK-919) OR (Sinopharm) OR (CoronaVac) OR ("Sputnik V") OR (Novavax) OR (NVX-CoV2373) OR (TAK-019) OR (SARS-CoV-2 rS) OR (NVX-CoV2601) OR ("COVID-19 vaccine") OR ("COVID-19 vaccines") OR ("COVID-19 vaccination")) AND (("Cross-sectional") OR (Cohort) OR (Intervention) OR (ecological) OR (Survey)) NOT ((Review) OR (Meta-analysis) OR ("Systematic Review"))**

Final result: 50,472 (PubMed- 22,798; Scopus- 15,717; Embase- 11,810; Cochrane Library- 147 articles (Filtered publication date 2009.01.01-2024.12.31)

Number of articles included in the systematic review: 264

**References (to 264 articles included in the systematic review)**


1. Freed GL, Clark SJ, Butchart AT, Singer DC, Davis MM. Parental vaccine safety concerns in 2009. Pediatrics. 2010 Apr;125(4):654-9. doi: 10.1542/peds.2009-1962.Epub 2010 Mar 1.

2. Ilter E, Celik A, Haliloglu B, et al. Women's knowledge of Pap smear test and human papillomavirus: acceptance of HPV vaccination to themselves and their daughters in an Islamic society. Int J Gynecol Cancer. 2010 Aug;20(6):1058-62. doi: 10.1111/IGC.0b013e3181dda2b9.

3. Flood EM, Rousculp MD, Ryan KJ, et al. Parents' decision-making regarding vaccinating their children against influenza: A web-based survey. Clin Ther. 2010 Aug;32(8):1448-67. doi: 10.1016/j.clinthera.2010.06.020.

4. Cheng PJ, Huang SY, Shaw SW, et al. Factors influencing women's decisions regarding pertussis vaccine: A decision-making study in the Postpartum Pertussis Immunization Program of a teaching hospital in Taiwan. Vaccine. 2010 Aug 2;28(34):5641-7. doi: 10.1016/j.vaccine.2010.05.078. Epub 2010 Jun 19.

5. Reiter PL, McRee AL, Gottlieb SL, Brewer NT. HPV vaccine for adolescent males: acceptability to parents post-vaccine licensure. Vaccine. 2010 Aug 31;28(38):6292-7. doi: 10.1016/j.vaccine.2010.06.114. Epub 2010 Jul 15.

6. Middleman AB, Tung JS. Urban middle school parent perspectives: the vaccines they are willing to have their children receive using school-based immunization programs. J Adolesc Health. 2010 Sep;47(3):249-53. doi: 10.1016/j.jadohealth.2010.01.009. Epub 2010 Apr 2.

7. Mortensen GL. Parental attitudes towards vaccinating sons with human papillomavirus vaccine. Dan Med Bull. 2010 Dec;57(12):A4230.

8. DiAngi YT, Panozzo CA, Ramogola-Masire D, Steenhoff AP, RCBrewer NT. A cross-sectional study of HPV vaccine acceptability in Gaborone, Botswana. PLoS One. 2011;6(10):e25481. doi: 10.1371/journal.pone.0025481. Epub 2011 Oct 25.

9. Brown KF, Shanley R, Cowley NA, et al. Attitudinal and demographic predictors of measles, mumps and rubella (MMR) vaccine acceptance: development and validation of an evidence-based measurement instrument. Vaccine. 2011 Feb 11;29(8):1700-9. doi: 10.1016/j.vaccine.2010.12.030. Epub 2010 Dec 24.

10. Kadis JA, McRee AL, Gottlieb SL, et al. Mothers' support for voluntary provision of HPV vaccine in schools. Vaccine. 2011 Mar 21;29(14):2542-7. doi: 10.1016/j.vaccine.2011.01.067. Epub 2011 Feb 5.

11. Kennedy A, Basket M, Sheedy K. Vaccine attitudes, concerns, and information sources reported by parents of young children: results from the 2009 HealthStyles survey. Pediatrics. 2011 May;127 Suppl 1:S92-9. doi: 10.1542/peds.2010-1722N. Epub 2011 Apr 18.

12. Charakorn C, Rattanasiri S, Lertkhachonsuk AA, Thanapprapasr D, Chittithaworn S, Wilailak S. Knowledge of Pap smear, HPV and the HPV vaccine and the acceptability of the HPV vaccine by Thai women. Asia Pac J Clin Oncol. 2011 Jun;7(2):160-7. doi: 10.1111/j.1743-7563.2011.01392.x.

13. Smith PJ, Humiston SG, Marcuse EK, et al. Parental delay or refusal of vaccine doses, childhood vaccination coverage at 24 months of age, and the Health Belief Model. Public Health Rep. 2011 Jul-Aug;126 Suppl 2(Suppl 2):135-46. doi: 10.1177/00333549111260S215.

14. Liu S, Yuan H, Liu Y, et al. Attitudes of seasonal influenza vaccination among healthcare worker and general community population after pandemic influenza A/H1N1 in Hangzhou. Hum Vaccin. 2011 Oct;7(10):1072-6. doi: 10.4161/hv.7.10.16781. Epub 2011 Oct 1.

15. Jaspers L, Budiningsih S, Wolterbeek R, Henderson FC, Peters AA. Parental acceptance of human papillomavirus (HPV) vaccination in Indonesia: a cross-sectional study. Vaccine. 2011 Oct 13;29(44):7785-93. doi: 10.1016/j.vaccine.2011.07.107. Epub 2011 Aug 5.

16. Marek E, Dergez T, Rebek-Nagy G, et al. Adolescents' awareness of HPV infections and attitudes towards HPV vaccination 3 years following the introduction of the HPV vaccine in Hungary. Vaccine. 2011 Nov 3;29(47):8591-8. doi: 10.1016/j.vaccine.2011.09.018. Epub 2011 Sep 21.

17. Zhao FH, Tiggelaar SM, Hu SY, et al. A multi-center survey of HPV knowledge and attitudes toward HPV vaccination among women, government officials, and medical personnel in China. Asian Pac J Cancer Prev. 2012;13(5):2369-78. doi: 10.7314/apjcp.2012.13.5.2369.

18. Hanley SJ, Yoshioka E, Ito Y, et al. Acceptance of and attitudes towards human papillomavirus vaccination in Japanese mothers of adolescent girls. Vaccine. 2012 Aug 24;30(39):5740-7. doi: 10.1016/j.vaccine.2012.07.003. Epub 2012 Jul 13.

19. Ayissi CA, Wamai RG, Oduwo GO, et al. Awareness, acceptability and uptake of human papilloma virus vaccine among Cameroonian school-attending female adolescents. J Community Health. 2012 Dec;37(6):1127-35. doi: 10.1007/s10900-012-9554-z.

20. Tonguc E, Gungor T, Var T, Kavak E, Yucel M, Uzunlar O. Knowledge about HPV, relation between HPV and cervix cancer and acceptance of HPV vaccine in women in eastern region of Turkey. J Gynecol Oncol. 2013 Jan;24(1):7-13. doi: 10.3802/jgo.2013.24.1.7. Epub 2013 Jan 8.

21. Wada K, Smith DR. Influenza vaccination uptake among the working age population of Japan: results from a national cross-sectional survey. PLoS One. 2013;8(3):e59272. doi: 10.1371/journal.pone.0059272. Epub 2013 Mar 12.

22. Lai JY, Tinker AV, Cheung WY. Factors influencing the willingness of US women to vaccinate their daughters against the human papillomavirus to prevent cervical cancer. Med Oncol. 2013;30(2):582. doi: 10.1007/s12032-013-0582-z. Epub 2013 Apr 23.

23. Tisi G, Salinaro F, Apostoli P, et al. HPV vaccination acceptability in young boys. Ann Ist Super Sanita. 2013;49(3):286-91. doi: 10.4415/ANN_13_03_09.

24. Kim IS, Seo YB, Hong KW, et al. Perceptions of Tetanus-diphteria-acellular pertussis (Tdap) Vaccination among Korean Women of Childbearing Age. Infect Chemother. 2013 Jun;45(2):217-24. doi: 10.3947/ic.2013.45.2.217. Epub 2013 Jun 26.

25. Zhang SK, Pan XF, Wang SM, et al. Perceptions and acceptability of HPV vaccination among parents of young adolescents: a multicenter national survey in China. Vaccine. 2013 Jul 11;31(32):3244-9. doi: 10.1016/j.vaccine.2013.05.046. Epub 2013 May 24.

26. Alder S, Perinetti C, Mints M, et al. Acceptance of human papillomavirus (HPV) vaccination among young women in a country with a high prevalence of HPV infection. Int J Oncol. 2013 Oct;43(4):1310-8. doi: 10.3892/ijo.2013.2023. Epub 2013 Jul 19.

27. Hanley SJ, Yoshioka E, Ito Y, et al. An exploratory study of Japanese fathers' knowledge of and attitudes towards HPV and HPV vaccination: does marital status matter?. Asian Pac J Cancer Prev. 2014;15(4):1837-43. doi: 10.7314/apjcp.2014.15.4.1837.

28. Masadeh MM, Alzoubi KH, Al-Azzam SI, Al-Agedi HS, Abu Rashid BE, Mukattash TL. Public awareness regarding children vaccination in Jordan. Hum Vaccin Immunother. 2014;10(6):1762-6. doi: 10.4161/hv.28608. Epub 2014 May 7.

29. Kruiroongroj S, Chaikledkaew U, Thavorncharoensap M. Knowledge, acceptance, and willingness to pay for human papilloma virus (HPV) vaccination among female parents in Thailand. Asian Pac J Cancer Prev. 2014;15(13):5469-74. doi: 10.7314/apjcp.2014.15.13.5469.

30. Johnson DC, Bhatta MP, Gurung S, Aryal S, Lhaki P, Shrestha S. Knowledge and awareness of human papillomavirus (HPV), cervical cancer and HPV vaccine among women in two distinct Nepali communities. Asian Pac J Cancer Prev. 2014;15(19):8287-93. doi: 10.7314/apjcp.2014.15.19.8287.

31. Madhivanan P, Li T, Srinivas V, Marlow L, Mukherjee S, Krupp K. Human papillomavirus vaccine acceptability among parents of adolescent girls: obstacles and challenges in Mysore, India. Prev Med. 2014 Jul;64:69-74. doi: 10.1016/j.ypmed.2014.04.002. Epub 2014 Apr 13.

32. Muhwezi WW, Banura C, Turiho AK, Mirembe F. Parents' knowledge, risk perception and willingness to allow young males to receive human papillomavirus (HPV) vaccines in Uganda. PLoS One. 2014 Sep 9;9(9):e106686. doi: 10.1371/journal.pone.0106686. eCollection 2014.

33. Vermandere H, Naanyu V, Mabeya H, Vanden Broeck D, Michielsen K, Degomme O. Determinants of acceptance and subsequent uptake of the HPV vaccine in a cohort in Eldoret, Kenya. PLoS One. 2014 Oct 9;9(10):e109353. doi: 10.1371/journal.pone.0109353. eCollection 2014.

34. Tsai YY, Lee JJ, Hsieh WH. Determinants of the public intent to receive the seasonal influenza vaccine and protective behaviors: a population-based study in Taiwan. Vaccine. 2014 Nov 20;32(49):6667-75. doi: 10.1016/j.vaccine.2014.08.080. Epub 2014 Sep 19.

35. Krawczyk A, Knäuper B, Gilca V, et al. Parents' decision-making about the human papillomavirus vaccine for their daughters: I. Quantitative results. Hum Vaccin Immunother. 2015;11(2):322-9. doi: 10.1080/21645515.2014.1004030.

36. Akanbi OA, Iyanda A, Osundare F, Opaleye OO. Perceptions of Nigerian Women about Human Papilloma Virus, Cervical Cancer, and HPV Vaccine. Scientifica (Cairo). 2015;2015:285702. doi: 10.1155/2015/285702. Epub 2015 Oct 13.

37. He L, Liao QY, Huang YQ, Feng S, Zhuang XM. Parents' perception and their decision on their children's vaccination against seasonal influenza in Guangzhou. Chin Med J (Engl). 2015 Feb 5;128(3):327-41. doi: 10.4103/0366-6999.150099.

38. Khurana S, Sipsma HL, Caskey RN. HPV vaccine acceptance among adolescent males and their parents in two suburban pediatric practices. Vaccine. 2015 Mar 24;33(13):1620-4. doi: 10.1016/j.vaccine.2015.01.038. Epub 2015 Feb 7.

39. Dempsey AF, Brewer SE, Pyrzanowski J, Sevick C, O'leary ST. Acceptability of human papillomavirus vaccines among women older than 26 years. Vaccine. 2015 Mar 24;33(13):1556-61. doi: 10.1016/j.vaccine.2015.02.018. Epub 2015 Feb 19.

40. Alder S, Gustafsson S, Perinetti C, Mints M, Sundström K, Andersson S. Mothers' acceptance of human papillomavirus (HPV) vaccination for daughters in a country with a high prevalence of HPV. Oncol Rep. 2015 May;33(5):2521-8. doi: 10.3892/or.2015.3817. Epub 2015 Feb 25.

41. Wang W, Ma Y, Wang X, et al. Acceptability of human papillomavirus vaccine among parents of junior middle school students in Jinan, China. Vaccine. 2015 May 21;33(22):2570-6. doi: 10.1016/j.vaccine.2015.04.010. Epub 2015 Apr 14.

42. Lee Mortensen G, Adam M, Idtaleb L. Parental attitudes towards male human papillomavirus vaccination: a pan-European cross-sectional survey. BMC Public Health. 2015 Jul 8;15:624. doi: 10.1186/s12889-015-1863-6.

43. Pavličević I, Škrabić S, Malički M, Merćep AH, Marušić M, Marušić A. Decisional conflict and vaccine uptake: cross-sectional study of 2012/2013 influenza season in Croatia. Arch Med Sci. 2015 Aug 12;11(4):788-95. doi: 10.5114/aoms.2015.53299. Epub 2015 Aug 11.

44. Moss JL, Reiter PL, Brewer NT. HPV vaccine for teen boys: Dyadic analysis of parents' and sons' beliefs and willingness. Prev Med. 2015 Sep;78:65-71. doi: 10.1016/j.ypmed.2015.07.002. Epub 2015 Jul 17.

45. Selmouni F, Zidouh A, Nejjari C, Bekkali R. Acceptability of the human papilloma virus vaccine among Moroccan parents: a population-based crosssectional study. East Mediterr Health J. 2015 Sep 28;21(8):555-63. doi: 10.26719/2015.21.8.555.

46. Morhason-Bello IO, Wallis S, Adedokun BO, Adewole IF. Willingness of reproductive-aged women in a Nigerian community to accept human papillomavirus vaccination for their children. J Obstet Gynaecol Res.

47. Maier C, Maier T, Neagu CE, Vlădăreanu R. Romanian adolescents' knowledge and attitudes towards human papillomavirus infection and prophylactic vaccination. Eur J Obstet Gynecol Reprod Biol. 2015 Dec;195:77-82. doi: 10.1016/j.ejogrb.2015.09.029. Epub 2015 Sep 30.

48. Dáňová J, Šálek J, Kocourková A, Čelko AM. Factors Associated with Parental Refusal of Routine Vaccination in the Czech Republic. Cent Eur J Public Health. 2015 Dec;23(4):321-3. doi: 10.21101/cejph.a4395.

49. Óskarsson Ý, Guðnason Þ, Jónsdóttir GA, Kristinsson KG, Briem H, Haraldsson Á. Public opinion on childhood immunisations in Iceland. Vaccine. 2015 Dec 16;33(51):7211-7216. doi: 10.1016/j.vaccine.2015.10.125. Epub 2015 Nov 10.

50. La Vincente SF, Mielnik D, Jenkins K, et al. Implementation of a national school-based Human Papillomavirus (HPV) vaccine campaign in Fiji: knowledge, vaccine acceptability and information needs of parents. BMC Public Health. 2015 Dec 18;15:1257. doi: 10.1186/s12889-015-2579-3.

51. Clark SJ, Cowan AE, Filipp SL, Fisher AM, Stokley S. Parent HPV vaccine perspectives and the likelihood of HPV vaccination of adolescent males. Hum Vaccin Immunother. 2016;12(1):47-51. doi: 10.1080/21645515.2015.1073426. Epub 2015 Jul 30.

52. Yu Y, Xu M, Sun J, et al. Human Papillomavirus Infection and Vaccination: Awareness and Knowledge of HPV and Acceptability of HPV Vaccine among Mothers of Teenage Daughters in Weihai, Shandong, China. PLoS One. 2016 Jan 14;11(1):e0146741. doi: 10.1371/journal.pone.0146741. eCollection 2016.

53. Voidăzan S, Tarcea M, Morariu SH, Grigore A, Dobreanu M. Human Papillomavirus Vaccine - Knowledge and Attitudes among Parents of Children Aged 10-14 Years: a Cross-sectional Study, Tîrgu Mureş, Romania. Cent Eur J Public Health. 2016 Mar;24(1):29-38. doi: 10.21101/cejph.a4287.

54. Dubé E, Gagnon D, Zhou Z, Deceuninck G. Parental Vaccine Hesitancy in Quebec (Canada). PLoS Curr. 2016 Mar 7;8:ecurrents.outbreaks.9e239605f4d320c6ad27ce2aea5aaad2. doi: 10.1371/currents.outbreaks.9e239605f4d320c6ad27ce2aea5aaad2.

55. Wang LD, Lam WW, Fielding R. Hong Kong Chinese parental attitudes towards vaccination and associated socio-demographic disparities. Vaccine. 2016 Mar 14;34(12):1426-9. doi: 10.1016/j.vaccine.2016.02.006. Epub 2016 Feb 8.

56. Gilbert NL, Gilmour H, Dubé È, Wilson SE, Laroche J. Estimates and determinants of HPV non-vaccination and vaccine refusal in girls 12 to 14 y of age in Canada: Results from the Childhood National Immunization Coverage Survey, 2013. Hum Vaccin Immunother. 2016 Jun 2;12(6):1484-90. doi: 10.1080/21645515.2016.1153207. Epub 2016 Mar 4.

57. Gilkey MB, McRee AL, Magnus BE, Reiter PL, Dempsey AF, Brewer NT. Vaccination Confidence and Parental Refusal/Delay of Early Childhood Vaccines. PLoS One. 2016 Jul 8;11(7):e0159087. doi: 10.1371/journal.pone.0159087. eCollection 2016.

58. Wong KK, Cohen AL, Norris SA, et al. Knowledge, attitudes, and practices about influenza illness and vaccination: a cross-sectional survey in two South African communities. Influenza Other Respir Viruses. 2016 Sep;10(5):421-8. doi: 10.1111/irv.12388. Epub 2016 Apr 29.

59. How CH, Phua See Chun P, Shafi F, Jakes RW. Parental knowledge, attitudes and perception of pneumococcal disease and pneumococcal conjugate vaccines in Singapore: a questionnaire-based assessment. BMC Public Health. 2016 Sep 2;16(1):923. doi: 10.1186/s12889-016-3597-5.

60. Frew PM, Fisher AK, Basket MM, et al. Changes in childhood immunization decisions in the United States: Results from 2012 & 2014 National Parental Surveys. Vaccine. 2016 Nov 4;34(46):5689-5696. doi: 10.1016/j.vaccine.2016.08.001. Epub 2016 Oct 6.

61. Lee KN, Chang KH, Cho SS, Park SH, Park ST. Attitudes Regarding HPV Vaccinations of Children among Mothers with Adolescent Daughters in Korea. J Korean Med Sci. 2017 Jan;32(1):130-134. doi: 10.3346/jkms.2017.32.1.130.

62. Chudowolska-Kiełkowska M, Gieroń-Kozina B, Gawałko M, Kądalska E, Henszel Ł. Knowledge and beliefs in a selected group of parents of children aged 2 and less concerning preventive vaccination. Przegl Epidemiol. 2017;71(4):583-593.

63. Gichane MW, Calo WA, McCarthy SH, Walmer KA, Boggan JC, Brewer NT. Human Papillomavirus Awareness in Haiti: Preparing for a National HPV Vaccination Program. J Pediatr Adolesc Gynecol. 2017 Feb;30(1):96-101. doi: 10.1016/j.jpag.2016.07.003. Epub 2016 Aug 4.

64. My C, Danchin M, Willaby HW, Pemberton S, Leask J. Parental attitudes, beliefs, behaviours and concerns towards childhood vaccinations in Australia: A national online survey. Aust Fam Physician. 2017 Mar;46(3):145-151.

65. Nowak GJ, Cacciatore MA. Parents' confidence in recommended childhood vaccinations: Extending the assessment, expanding the context. Hum Vaccin Immunother. 2017 Mar 4;13(3):687-700. doi: 10.1080/21645515.2016.1236881. Epub 2016 Sep 28.

66. Campbell H, Edwards A, Letley L, Bedford H, Ramsay M, Yarwood J. Changing attitudes to childhood immunisation in English parents. Vaccine. 2017 May 19;35(22):2979-2985. doi: 10.1016/j.vaccine.2017.03.089. Epub 2017 Apr 23.

67. Boes L, Boedeker B, Schmich P, Wetzstein M, Wichmann O, Remschmidt C. Factors associated with parental acceptance of seasonal influenza vaccination for their children - A telephone survey in the adult population in Germany. Vaccine. 2017 Jun 27;35(30):3789-3796. doi: 10.1016/j.vaccine.2017.05.015. Epub 2017 May 27.

68. Lee S, Riley-Behringer M, Rose JC, Meropol SB, Lazebnik R. Parental Vaccine Acceptance: A Logistic Regression Model Using Previsit Decisions. Clin Pediatr (Phila). 2017 Jul;56(8):716-722. doi: 10.1177/0009922816670980. Epub 2016 Oct 26.

69. Schollin Ask L, Hjern A, Lindstrand A, et al. Receiving early information and trusting Swedish child health centre nurses increased parents' willingness to vaccinate against rotavirus infections. Acta Paediatr. 2017 Aug;106(8):1309-1316. doi: 10.1111/apa.13872. Epub 2017 Jun 1.

70. Loke AY, Chan ACO, Wong YT. Facilitators and barriers to the acceptance of human papillomavirus (HPV) vaccination among adolescent girls: a comparison between mothers and their adolescent daughters in Hong Kong. BMC Res Notes. 2017 Aug 10;10(1):390. doi: 10.1186/s13104-017-2734-2.

71. Low MSF, Tan H, Hartman M, et al. Parental perceptions of childhood seasonal influenza vaccination in Singapore: A cross-sectional survey. Vaccine. 2017 Oct 27;35(45):6096-6102. doi: 10.1016/j.vaccine.2017.09.060. Epub 2017 Sep 27.

72. Lee CHJ, Duck IM, Sibley CG. Personality and demographic correlates of New Zealanders' confidence in the safety of childhood vaccinations. Vaccine. 2017 Oct 27;35(45):6089-6095. doi: 10.1016/j.vaccine.2017.09.061. Epub 2017 Sep 28.

73. SteelFisher GK, Blendon RJ, Guirguis S, et al. Understanding threats to polio vaccine commitment among caregivers in high-priority areas of Afghanistan: a polling study. Lancet Infect Dis. 2017 Nov;17(11):1172-1179. doi: 10.1016/S1473-3099(17)30397-3. Epub 2017 Aug 14.

74. Masters NB, Tefera YA, Wagner AL, Boulton ML. Vaccine hesitancy among caregivers and association with childhood vaccination timeliness in Addis Ababa, Ethiopia. Hum Vaccin Immunother. 2018;14(10):2340-2347. doi: 10.1080/21645515.2018.1480242. Epub 2018 Jun 28.

75. Alshammari TM, Subaiea GM, Hussain T, Moin A, Yusuff KB. Parental perceptions, attitudes and acceptance of childhood immunization in Saudi Arabia: A cross sectional study. Vaccine. 2018 Jan 2;36(1):23-28. doi: 10.1016/j.vaccine.2017.11.050. Epub 2017 Nov 22.

76. Dubé E, Gagnon D, Ouakki M, et al. Measuring vaccine acceptance among Canadian parents: A survey of the Canadian Immunization Research Network. Vaccine. 2018 Jan 25;36(4):545-552. doi: 10.1016/j.vaccine.2017.12.005. Epub 2017 Dec 9.

77. Giambi C, Fabiani M, D'Ancona F, et al. Parental vaccine hesitancy in Italy - Results from a national survey. Vaccine. 2018 Feb 1;36(6):779-787. doi: 10.1016/j.vaccine.2017.12.074. Epub 2018 Jan 8.

78. Ganczak M, Owsianka B, Korzeń M. Factors that Predict Parental Willingness to Have Their Children Vaccinated against HPV in a Country with Low HPV Vaccination Coverage. Int J Environ Res Public Health. 2018 Mar 31;15(4):645. doi: 10.3390/ijerph15040645.

79. Rey D, Fressard L, Cortaredona S, et al. Vaccine hesitancy in the French population in 2016, and its association with vaccine uptake and perceived vaccine risk-benefit balance. Euro Surveill. 2018 Apr;23(17):17-00816. doi: 10.2807/1560-7917.ES.2018.23.17.17-00816.

80. Sherman SM, Nailer E. Attitudes towards and knowledge about Human Papillomavirus (HPV) and the HPV vaccination in parents of teenage boys in the UK. PLoS One. 2018 Apr 11;13(4):e0195801. doi: 10.1371/journal.pone.0195801. eCollection 2018.

81. Endarti D, Satibi S, Kristina SA, Farida MA, Rahmawanti Y, Andriani T. Knowledge, Perception, and Acceptance of HPV Vaccination and Screening for Cervical Cancer among Women in

Yogyakarta Province, Indonesia. Asian Pac J Cancer Prev. 2018 Apr 27;19(4):1105-1111. doi: 10.22034/APJCP.2018.19.4.1105.

82. Wang Z, Wang J, Fang Y, et al. Parental acceptability of HPV vaccination for boys and girls aged 9-13 years in China - A population-based study. Vaccine. 2018 May 3;36(19):2657-2665. doi: 10.1016/j.vaccine.2018.03.057. Epub 2018 Mar 30.

83. Bardají A, Mindu C, Augusto OJ, et al. Awareness of cervical cancer and willingness to be vaccinated against human papillomavirus in Mozambican adolescent girls. Papillomavirus Res. 2018 Jun;5:156-162. doi: 10.1016/j.pvr.2018.04.004. Epub 2018 Apr 14.

84. Pelullo CP, Di Giuseppe G. Vaccinations among Italian adolescents: Knowledge, attitude and behavior. Hum Vaccin Immunother. 2018 Jul 3;14(7):1566-1572. doi: 10.1080/21645515.2017.1421877. Epub 2018 Jan 25.

85. Napolitano F, D'Alessandro A, Angelillo IF. Investigating Italian parents' vaccine hesitancy: A cross-sectional survey. Hum Vaccin Immunother. 2018 Jul 3;14(7):1558-1565. doi: 10.1080/21645515.2018.1463943. Epub 2018 May 14.

86. Marić G, Birčanin Đ, Kisić V, et al. Parental perspective on human papillomavirus (HPV) vaccination in Serbia: Knowledge, attitudes and practice. Sex Reprod Healthc. 2018 Jun;16:192-198. doi: 10.1016/j.srhc.2018.04.010. Epub 2018 Apr 17.

87. Domek GJ, O'Leary ST, Bull S, et al. Measuring vaccine hesitancy: Field testing the WHO SAGE Working Group on Vaccine Hesitancy survey tool in Guatemala. Vaccine. 2018 Aug 23;36(35):5273-5281. doi: 10.1016/j.vaccine.2018.07.046. Epub 2018 Jul 27.

88. Juntasopeepun P, Thana K. Parental acceptance of HPV vaccines in Chiang Mai, Thailand. Int J Gynaecol Obstet. 2018 Sep;142(3):343-348. doi: 10.1002/ijgo.12539. Epub 2018 Jun 21.

89. Učakar V, Fafangel M, Kraigher A. Vaccine confidence among mothers of young children, Slovenia, 2016. Vaccine. 2018 Sep 5;36(37):5544-5550. doi: 10.1016/j.vaccine.2018.07.062. Epub 2018 Aug 1.

90. Mendes Lobão W, Duarte FG, Burns JD, et al. Low coverage of HPV vaccination in the national immunization programme in Brazil: Parental vaccine refusal or barriers in health-service based vaccine delivery?. PLoS One. 2018 Nov 12;13(11):e0206726. doi: 10.1371/journal.pone.0206726. eCollection 2018.

91. Lovrić Makarić Z, Kolarić B, Tomljenović M, Posavec M. Attitudes and beliefs related to childhood vaccinations among parents of 6 years old children in Zagreb, Croatia. Vaccine. 2018 Nov 26;36(49):7530-7535. doi: 10.1016/j.vaccine.2018.10.055. Epub 2018 Oct 23.

92. Degarege A, Krupp K, Fennie K, et al. Human Papillomavirus Vaccine Acceptability among Parents of Adolescent Girls in a Rural Area, Mysore, India. J Pediatr Adolesc Gynecol. 2018 Dec;31(6):583-591. doi: 10.1016/j.jpag.2018.07.008. Epub 2018 Jul 26.

93. Dubé È, Farrands A, Lemaitre T, et al. Overview of knowledge, attitudes, beliefs, vaccine hesitancy and vaccine acceptance among mothers of infants in Quebec, Canada. Hum Vaccin Immunother. 2019;15(1):113-120. doi: 10.1080/21645515.2018.1509647. Epub 2018 Sep 11.

94. Hu Y, Chen Y, Wang Y, Liang H. Measuring childhood vaccination acceptance of mother in Zhejiang province, East China. Hum Vaccin Immunother. 2019;15(2):287-294. doi: 10.1080/21645515.2018.1526557. Epub 2018 Oct 5.

95. Guay M, Gosselin V, Petit G, Baron G, Gagneur A. Determinants of vaccine hesitancy in Quebec: a large population-based survey. Hum Vaccin Immunother. 2019;15(11):2527-2533. doi: 10.1080/21645515.2019.1603563. Epub 2019 May 17.

96. Balbir Singh HK, Badgujar VB, Yahaya RS, et al. Assessment of knowledge and attitude among postnatal mothers towards childhood vaccination in Malaysia. Hum Vaccin Immunother. 2019;15(11):2544-2551. doi: 10.1080/21645515.2019.1612666. Epub 2019 Jun 20.

97. Bianco A, Mascaro V, Zucco R, Pavia M. Parent perspectives on childhood vaccination: How to deal with vaccine hesitancy and refusal?. Vaccine. 2019 Feb 8;37(7):984-990. doi: 10.1016/j.vaccine.2018.12.062. Epub 2019 Jan 14.

98. Rossen I, Hurlstone MJ, Dunlop PD, Lawrence C. Accepters, fence sitters, or rejecters: Moral profiles of vaccination attitudes. Soc Sci Med. 2019 Mar;224:23-27. doi: 10.1016/j.socscimed.2019.01.038. Epub 2019 Jan 29.

99. Zeng Y, Yuan Z, Yin J, Han Y, Chu CI, Fang Y. Factors affecting parental intention to vaccinate kindergarten children against influenza: A cross-sectional survey in China. Vaccine. 2019 Mar 7;37(11):1449-1456. doi: 10.1016/j.vaccine.2019.01.071. Epub 2019 Feb 12.

100. Luyten J, Bruyneel L, van Hoek AJ. Assessing vaccine hesitancy in the UK population using a generalized vaccine hesitancy survey instrument. Vaccine. 2019 Apr 24;37(18):2494-2501. doi: 10.1016/j.vaccine.2019.03.041. Epub 2019 Mar 30.

101. Suzuki Y, Sukegawa A, Nishikawa A, et al. Current knowledge of and attitudes toward human papillomavirus-related disease prevention among Japanese: A large-scale questionnaire study. J Obstet Gynaecol Res. 2019 May;45(5):994-1005. doi: 10.1111/jog.13929. Epub 2019 Feb 5.

102. Han Y, Yin J, Zeng Y, Chu CI, Chiang YC, Fang Y. Determinants of Parental Intentions to Vaccinate Kindergarten Children Against Seasonal Influenza in Xiamen, China. J Prim Prev. 2019 Jun;40(3):325-342. doi: 10.1007/s10935-019-00550-7.

103. Napolitano F, Della Polla G, Angelillo IF. Knowledge, Attitudes, and Behaviors of Parents towards Recommended Adult Vaccinations: An Explanatory Survey in the Geographic Area of Naples, Italy. Int J Environ Res Public Health. 2019 Jun 12;16(12):2070. doi: 10.3390/ijerph16122070.

104. Lin W, Wang Y, Liu Z, et al. Awareness and attitude towards human papillomavirus and its vaccine among females with and without daughter(s) who participated in cervical cancer screening in Shenzhen, China. Trop Med Int Health. 2019 Sep;24(9):1054-1063. doi: 10.1111/tmi.13283. Epub 2019 Jul 24.

105. Alsubaie SS, Gosadi IM, Alsaadi BM, et al. Vaccine hesitancy among Saudi parents and its determinants. Result from the WHO SAGE working group on vaccine hesitancy survey tool. Saudi Med J. 2019 Dec;40(12):1242-1250. doi: 10.15537/smj.2019.12.24653.

106. Krishnamoorthy Y, Kannusamy S, Sarveswaran G, Majella MG, Sarkar S, Narayanan V. Factors related to vaccine hesitancy during the implementation of Measles-Rubella campaign 2017 in rural

Puducherry-A mixed-method study. J Family Med Prim Care. 2019 Dec 10;8(12):3962-3970. doi: 10.4103/jfmpc.jfmpc_790_19. eCollection 2019 Dec.

107. Lin W, Wang Y, Liu Z, et al. Inequalities in Awareness and Attitude towards HPV and Its Vaccine between Local and Migrant Residents Who Participated in Cervical Cancer Screening in Shenzhen, China. Cancer Res Treat. 2020 Jan;52(1):207-217. doi: 10.4143/crt.2019.053. Epub 2019 Jul 1.

108. Hadjipanayis A, van Esso D, Del Torso S, et al. Vaccine confidence among parents: Large scale study in eighteen European countries. Vaccine. 2020 Feb 5;38(6):1505-1512. doi: 10.1016/j.vaccine.2019.11.068. Epub 2019 Dec 14.

109. Yufika A, Wagner AL, Nawawi Y, et al. Parents' hesitancy towards vaccination in Indonesia: A cross-sectional study in Indonesia. Vaccine. 2020 Mar 4;38(11):2592-2599. doi: 10.1016/j.vaccine.2020.01.072. Epub 2020 Feb 1.

110. Lin Y, Lin Z, He F, et al. HPV vaccination intent and willingness to pay for 2-,4-, and 9-valent HPV vaccines: A study of adult women aged 27-45 years in China. Vaccine. 2020 Mar 23;38(14):3021-3030. doi: 10.1016/j.vaccine.2020.02.042. Epub 2020 Feb 29.

111. Byström E, Lindstrand A, Bergström J, Riesbeck K, Roth A. Confidence in the National Immunization Program among parents in Sweden 2016 - A cross-sectional survey. Vaccine. 2020 May 8;38(22):3909-3917. doi: 10.1016/j.vaccine.2020.01.078. Epub 2020 Feb 11.

112. Lewandowska A, Lewandowski T, Rudzki G, Rudzki S, Laskowska B. Opinions and Knowledge of Parents Regarding Preventive Vaccinations of Children and Causes of Reluctance toward Preventive Vaccinations. Int J Environ Res Public Health. 2020 May 24;17(10):3694. doi: 10.3390/ijerph17103694.

113. Wagner AL, Gordon A, Tallo VL, et al. Intent to obtain pediatric influenza vaccine among mothers in four middle income countries. Vaccine. 2020 Jun 2;38(27):4325-4335. doi: 10.1016/j.vaccine.2020.04.028. Epub 2020 May 6.

114. Bertoncello C, Ferro A, Fonzo M, et al. Socioeconomic Determinants in Vaccine Hesitancy and Vaccine Refusal in Italy. Vaccines (Basel). 2020 Jun 5;8(2):276. doi: 10.3390/vaccines8020276.

115. Lee CHJ, Sibley CG. Attitudes toward vaccinations are becoming more polarized in New Zealand: Findings from a longitudinal survey. EClinicalMedicine. 2020 Jun 6;23:100387. doi: 10.1016/j.eclinm.2020.100387. eCollection 2020 Jun.

116. Steens A, Stefanoff P, Daae A, Vestrheim DF, Riise Bergsaker MA. High overall confidence in childhood vaccination in Norway, slightly lower among the unemployed and those with a lower level of education. Vaccine. 2020 Jun 15;38(29):4536-4541. doi: 10.1016/j.vaccine.2020.05.011. Epub 2020 May 21.

117. Akinleye HW, Kanma-Okafor OJ, Okafor IP, Odeyemi KA. Parental willingness to vaccinate adolescent daughters against human papilloma virus for cervical cancer prevention in Western Nigeria. Pan Afr Med J. 2020 Jun 19;36:112. doi: 10.11604/pamj.2020.36.112.19007. eCollection 2020.

118. Rezaei S, Woldemichael A, Mirzaei M, Mohammadi S, Karami Matin B. Mothers' willingness to accept and pay for vaccines to their children in western Iran: a contingent valuation study. BMC Pediatr. 2020 Jun 23;20(1):307. doi: 10.1186/s12887-020-02208-4.

119. Furman FM, Zgliczyński WS, Jankowski M, Baran T, Szumowski Ł, Pinkas J. The State of Vaccine Confidence in Poland: A 2019 Nationwide Cross-Sectional Survey. Int J Environ Res Public Health. 2020 Jun 24;17(12):4565. doi: 10.3390/ijerph17124565.

120. Migriño J Jr, Gayados B, Birol KRJ, et al. Factors affecting vaccine hesitancy among families with children 2 years old and younger in two urban communities in Manila, Philippines. Western Pac Surveill Response J. 2020 Jun 30;11(2):20-26. doi: 10.5365/wpsar.2019.10.2.006. eCollection 2020 Apr-Jun.

121. Kempe A, Saville AW, Albertin C, et al. Parental Hesitancy About Routine Childhood and Influenza Vaccinations: A National Survey. Pediatrics. 2020 Jul;146(1):e20193852. doi: 10.1542/peds.2019-3852. Epub 2020 Jun 15.

122. Szilagyi PG, Albertin CS, Gurfinkel D, et al. Prevalence and characteristics of HPV vaccine hesitancy among parents of adolescents across the US. Vaccine. 2020 Aug 27;38(38):6027-6037. doi: 10.1016/j.vaccine.2020.06.074. Epub 2020 Aug 2.

123. Tu S, Sun FY, Chantler T, et al. Caregiver and service provider vaccine confidence following the Changchun Changsheng vaccine incident in China: A cross-sectional mixed methods study. Vaccine. 2020 Oct 14;38(44):6882-6888. doi: 10.1016/j.vaccine.2020.08.063. Epub 2020 Sep 8.

124. Barry MA, Aljammaz KI, Alrashed AA. Knowledge, Attitude, and Barriers Influencing Seasonal Influenza Vaccination Uptake. Can J Infect Dis Med Microbiol. 2020 Oct 16;2020:7653745. doi: 10.1155/2020/7653745. eCollection 2020.

125. Huon JF, Grégoire A, Meireles A, et al. Evaluation of the acceptability in France of the vaccine against papillomavirus (HPV) among middle and high school students and their parents. PLoS One. 2020 Oct 22;15(10):e0234693. doi: 10.1371/journal.pone.0234693. eCollection 2020.

126. Alawneh I, Saymeh A, Yasin A, Alawneh M, Al-Tatari H. Vaccines Attitudes, Concerns, and Information Sources Reported by Parents of Young Children among North Palestinian Parents. Adv Prev Med. 2020 Oct 31;2020:8028172. doi: 10.1155/2020/8028172. eCollection 2020.

127. Charron J, Gautier A, Jestin C. Influence of information sources on vaccine hesitancy and practices. Med Mal Infect. 2020 Nov;50(8):727-733. doi: 10.1016/j.medmal.2020.01.010. Epub 2020 Feb 14.

128. Della Polla G, Pelullo CP, Napolitano F, Angelillo IF. HPV vaccine hesitancy among parents in Italy: a cross-sectional study. Hum Vaccin Immunother. 2020 Nov 1;16(11):2744-2751. doi: 10.1080/21645515.2020.1744367. Epub 2020 Apr 16.

129. Du F, Chantler T, Francis MR, et al. The determinants of vaccine hesitancy in China: A cross-sectional study following the Changchun Changsheng vaccine incident. Vaccine. 2020 Nov 3;38(47):7464-7471. doi: 10.1016/j.vaccine.2020.09.075. Epub 2020 Oct 9.

130. Domnich A, Cambiaggi M, Vasco A, et al. Attitudes and Beliefs on Influenza Vaccination during the COVID-19 Pandemic: Results from a Representative Italian Survey. Vaccines (Basel). 2020 Nov 30;8(4):711. doi: 10.3390/vaccines8040711.

131. Alsuwaidi AR, Elbarazi I, Al-Hamad S, Aldhaheri R, Sheek-Hussein M, Narchi H. Vaccine hesitancy and its determinants among Arab parents: a cross-sectional survey in the United Arab Emirates. Hum Vaccin Immunother. 2020 Dec 1;16(12):3163-3169. doi: 10.1080/21645515.2020.1753439. Epub 2020 May 13.

132. Sokol RL, Grummon AH. COVID-19 and Parent Intention to Vaccinate Their Children Against Influenza. Pediatrics. 2020 Dec;146(6):e2020022871. doi: 10.1542/peds.2020-022871. Epub 2020 Sep 30.

133. Santibanez TA, Nguyen KH, Greby SM, et al. Parental Vaccine Hesitancy and Childhood Influenza Vaccination. Pediatrics. 2020 Dec;146(6):e2020007609. doi: 10.1542/peds.2020-007609. Epub 2020 Nov 9.

134. Bell S, Clarke R, Paterson P, Mounier-Jack S. Parents' and guardians' views and experiences of accessing routine childhood vaccinations during the coronavirus (COVID-19) pandemic: A mixed methods study in England. PLoS One. 2020 Dec 28;15(12):e0244049. doi: 10.1371/journal.pone.0244049. eCollection 2020.

135. Lin Y, Su Z, Chen F, et al. Chinese mothers' intention to vaccinate daughters against human papillomavirus (HPV), and their vaccine preferences: a study in Fujian Province. Hum Vaccin Immunother. 2021 Jan 2;17(1):304-315. doi: 10.1080/21645515.2020.1756152. Epub 2020 May 13.

136. Hamadah RE, Hussain AN, Alsoghayer NA, Alkhenizan ZA, Alajlan HA, Alkhenizan AH. Attitude of parents towards seasonal influenza vaccination for children in Saudi Arabia. J Family Med Prim Care. 2021 Feb;10(2):904-909. doi: 10.4103/jfmpc.jfmpc_1602_20. Epub 2021 Feb 27.

137. Mabeya H, Odunga J, Broeck DV. Mothers of adolescent girls and Human Papilloma Virus (HPV) vaccination in Western Kenya. Pan Afr Med J. 2021 Feb 4;38:126. doi: 10.11604/pamj.2021.38.126.21359. eCollection 2021.

138. Gerussi V, Peghin M, Palese A, et al. Vaccine Hesitancy among Italian Patients Recovered from COVID-19 Infection towards Influenza and Sars-Cov-2 Vaccination. Vaccines (Basel). 2021 Feb 18;9(2):172. doi: 10.3390/vaccines9020172.

139. Milondzo T, Meyer JC, Dochez C, Burnett RJ. Misinformation Drives Low Human Papillomavirus Vaccination Coverage in South African Girls Attending Private Schools. Front Public Health. 2021 Feb 19;9:598625. doi: 10.3389/fpubh.2021.598625. eCollection 2021.

140. Du F, Chantler T, Francis MR, et al. Access to Vaccination Information and Confidence/Hesitancy Towards Childhood Vaccination: A Cross-Sectional Survey in China. Vaccines (Basel). 2021 Feb 28;9(3):201. doi: 10.3390/vaccines9030201.

141. Mizumachi K, Aoki H, Kitano T, Onishi T, Takeyama M, Shima M. How to recover lost vaccine acceptance? A multi-center survey on HPV vaccine acceptance in Japan. J Infect Chemother. 2021 Mar;27(3):445-449. doi: 10.1016/j.jiac.2020.10.012. Epub 2020 Oct 27.

142. Khattak FA, Rehman K, Shahzad M, et al. Prevalence of Parental refusal rate and its associated factors in routine immunization by using WHO Vaccine Hesitancy tool: A Cross sectional study at district Bannu, KP, Pakistan. Int J Infect Dis. 2021 Mar;104:117-124. doi: 10.1016/j.ijid.2020.12.029. Epub 2020 Dec 17.

143. Thapar R, Kumar N, Surendran P, et al. Vaccine hesitancy among mothers of under-five children in Coastal South India: a facility-based cross-sectional study. F1000Res. 2021 Mar 8;10:186. doi: 10.12688/f1000research.28293.2. eCollection 2021.

144. Chung-Delgado K, Valdivia Venero JE, Vu TM. Vaccine Hesitancy: Characteristics of the Refusal of Childhood Vaccination in a Peruvian Population. Cureus. 2021 Mar 25;13(3):e14105. doi: 10.7759/cureus.14105.

145. Huang Y, Xu S, Xu Y, et al. A New Strategy for Cervical Cancer Prevention Among Chinese Women: How Much Do They Know and How Do They React Toward the HPV Immunization?. J Cancer Educ. 2021 Apr;36(2):386-394. doi: 10.1007/s13187-019-01642-y.

146. Fonseca IC, Pereira AI, Barros L. Portuguese parental beliefs and attitudes towards vaccination. Health Psychol Behav Med. 2021 May 6;9(1):422-435. doi: 10.1080/21642850.2021.1920948.

147. GebreEyesus FA, Tarekegn TT, Amlak BT, et al. Knowledge, Attitude, and Practices of Parents About Immunization of Infants and Its Associated Factors in Wadla Woreda, North East Ethiopia, 2019. Pediatric Health Med Ther. 2021 May 10;12:223-238. doi: 10.2147/PHMT.S295378. eCollection 2021.

148. Hou Z, Song S, Du F, et al. The Influence of the COVID-19 Epidemic on Prevention and Vaccination Behaviors Among Chinese Children and Adolescents: Cross-sectional Online Survey Study. JMIR Public Health Surveill. 2021 May 26;7(5):e26372. doi: 10.2196/26372.

149. Holroyd TA, Howa AC, Delamater PL, et al. Parental vaccine attitudes, beliefs, and practices: initial evidence in California after a vaccine policy change. Hum Vaccin Immunother. 2021 Jun 3;17(6):1675-1680. doi: 10.1080/21645515.2020.1839293. Epub 2020 Nov 24.

150. Tang X, Tang T, Yan R, Zhou Y, Deng X, He H. Intention to accept pertussis vaccination among Chinese people older than age 5. Hum Vaccin Immunother. 2021 Jun 3;17(6):1686-1692. doi: 10.1080/21645515.2020.1849517. Epub 2021 Jan 15.

151. Bauer A, Tiefengraber D, Wiedermann U. Towards understanding vaccine hesitancy and vaccination refusal in Austria. Wien Klin Wochenschr. 2021 Jul;133(13-14):703-713. doi: 10.1007/s00508-020-01777-9. Epub 2020 Dec 11.

152. Hendaus MA, Hassan M, Alsulaiti M, et al. Parents attitudes toward the human papilloma virus (HPV) vaccine: A new concept in the State of Qatar. J Family Med Prim Care. 2021 Jul;10(7):2488-2493. doi: 10.4103/jfmpc.jfmpc_1122_20. Epub 2021 Jul 30.

153. Gentile A, Pacchiotti AC, Giglio N, et al. Vaccine hesitancy in Argentina: Validation of WHO scale for parents. Vaccine. 2021 Jul 30;39(33):4611-4619. doi: 10.1016/j.vaccine.2021.06.080. Epub 2021 Jul 5.

154. Whelan SO, Moriarty F, Lawlor L, Gorman KM, Beamish J. Vaccine hesitancy and reported non-vaccination in an Irish pediatric outpatient population. Eur J Pediatr. 2021 Sep;180(9):2839-2847. doi: 10.1007/s00431-021-04039-6. Epub 2021 Mar 27.

155. Gkentzi D, Tsagri C, Kostopoulou E, et al. Attitudes and beliefs of parents about routine childhood vaccination in Greece. Hum Vaccin Immunother. 2021 Sep 2;17(9):3066-3072. doi: 10.1080/21645515.2021.1914805. Epub 2021 May 10.

156. Kyprianidou M, Tzira E, Galanis P, Giannakou K. Knowledge of mothers regarding children's vaccinations in Cyprus: A cross-sectional study. PLoS One. 2021 Sep 20;16(9):e0257590. doi: 10.1371/journal.pone.0257590. eCollection 2021.

157. Mayerová D, Abbas K. Childhood immunisation timeliness and vaccine confidence by health information source, maternal, socioeconomic, and geographic characteristics in Albania. BMC Public Health. 2021 Sep 22;21(1):1724. doi: 10.1186/s12889-021-11724-6.

158. Xie Y, Su LY, Wang F, Tang HY, Yang QG, Liu YJ. Awareness regarding and vaccines acceptability of human papillomavirus among parents of middle school students in Zunyi, Southwest China. Hum Vaccin Immunother. 2021 Nov 2;17(11):4406-4411. doi: 10.1080/21645515.2021.1951931. Epub 2021 Jul 29.

159. Yörük S, Güler D. Factors associated with pediatric vaccine hesitancy of parents: a cross-sectional study in Turkey. Hum Vaccin Immunother. 2021 Nov 2;17(11):4505-4511. doi: 10.1080/21645515.2021.1953348. Epub 2021 Oct 19.

160. Hussain A, Zahid A, Malik M, et al. Assessment of Parents' Perceptions of Childhood Immunization: A Cross-Sectional Study from Pakistan. Children (Basel). 2021 Nov 4;8(11):1007. doi: 10.3390/children8111007.

161. Adedire EB, Ajumobi O, Bolu O, Nguku P, Ajayi I. Maternal knowledge, attitude, and perception about childhood routine immunization program in Atakumosa-west Local Government Area, Osun State, Southwestern Nigeria. Pan Afr Med J. 2021 Nov 12;40(Suppl 1):8. doi: 10.11604/pamj.supp.2021.40.1.30876. eCollection 2021.

162. Samel-Kowalik P, Jankowski M, Lisiecka-Biełanowicz M, et al. Factors Associated with Attitudes towards Seasonal Influenza Vaccination in Poland: A Nationwide Cross-Sectional Survey in 2020. Vaccines (Basel). 2021 Nov 17;9(11):1336. doi: 10.3390/vaccines9111336.

163. Voo JYH, Lean QY, Ming LC, Md Hanafiah NH, Al-Worafi YM, Ibrahim B. Vaccine Knowledge, Awareness and Hesitancy: A Cross Sectional Survey among Parents Residing at Sandakan District, Sabah. Vaccines (Basel). 2021 Nov 17;9(11):1348. doi: 10.3390/vaccines9111348.

164. Saeed R, Hashmi I. Pakistan Ranks Third Globally With the Most Unvaccinated Children: Is the Impact of Parental Perception and Attitude on Immunization an Essential Contributing Factor to an Unsuccessful Vaccination Coverage?. Cureus. 2021 Nov 19;13(11):e19751. doi: 10.7759/cureus.19751. eCollection 2021 Nov.

165. Marshall S, Moore AC, Sahm LJ, Fleming A. Parent Attitudes about Childhood Vaccines: Point Prevalence Survey of Vaccine Hesitancy in an Irish Population. Pharmacy (Basel). 2021 Nov 23;9(4):188. doi: 10.3390/pharmacy9040188.

166. Zhang H, Zheng P, Zhang J, et al. Vaccine hesitancy among parents and its influencing factors: a cross-sectional study in Guangzhou, China. Hum Vaccin Immunother. 2021 Dec 2;17(12):5153-5161. doi: 10.1080/21645515.2021.1984131. Epub 2021 Dec 10.

167. Zibako P, Tsikai N, Manyame S, Ginindza TG. Knowledge, attitude and practice towards cervical cancer prevention among mothers of girls aged between 9 and 14 years: a cross sectional survey in Zimbabwe. BMC Womens Health. 2021 Dec 20;21(1):426. doi: 10.1186/s12905-021-01575-z.

168. Sabahelzain MM, Moukhyer M, Bosma H, van den Borne B. Determinants of Measles Vaccine Hesitancy among Sudanese Parents in Khartoum State, Sudan: A Cross-Sectional Study. Vaccines (Basel). 2021 Dec 22;10(1):6. doi: 10.3390/vaccines10010006.

169. Fadda G, Biasio LR, Mariani T, Giambi C. A survey about the degree of information and awareness of adolescents regarding vaccination in a Province of Central Italy. Ann Ig. 2022 Jan-Feb;34(1):13-26. doi: 10.7416/ai.2021.2457. Epub 2021 Jun 11.

170. Zalloum WA, Elayeh ER, Ali BAH, Zalloum N. Perception, knowledge and attitude towards influenza vaccine during COVID-19 pandemic in Jordanian population. Eur J Integr Med. 2022 Jan;49:102100. doi: 10.1016/j.eujim.2022.102100. Epub 2022 Jan 8.

171. Sabahelzain MM, Moukhyer M, van den Borne B, Bosma H. Vaccine Hesitancy among Parents and Its Association with the Uptake of Measles Vaccine in Urban Settings in Khartoum State, Sudan. Vaccines (Basel). 2022 Jan 28;10(2):205. doi: 10.3390/vaccines10020205.

172. Nguyen KH, Srivastav A, Lindley MC, et al. Parental Vaccine Hesitancy and Association With Childhood Diphtheria, Tetanus Toxoid, and Acellular Pertussis; Measles, Mumps, and Rubella; Rotavirus; and Combined 7-Series Vaccination. Am J Prev Med. 2022 Mar;62(3):367-376. doi: 10.1016/j.amepre.2021.08.015. Epub 2021 Dec 8.

173. Skitarelić N, Vidaić M, Skitarelić N. Parents' versus Grandparents' Attitudes about Childhood Vaccination. Children (Basel). 2022 Mar 2;9(3):345. doi: 10.3390/children9030345.

174. Cherian V, Saini NK, Sharma AK, Philip J. Prevalence and predictors of vaccine hesitancy in an urbanized agglomeration of New Delhi, India. J Public Health (Oxf). 2022 Mar 7;44(1):70-76. doi: 10.1093/pubmed/fdab007.

175. Banik R, Naher S, Rahman M, Gozal D. Investigating Bangladeshi Rural Women's Awareness and Knowledge of Cervical Cancer and Attitude Towards HPV Vaccination: a Community-Based Cross-Sectional Analysis. J Cancer Educ. 2022 Apr;37(2):449-460. doi: 10.1007/s13187-020-01835-w.

176. Galvão MPSP, Araújo TME, Rocha SSD. Knowledge, attitudes, and practices of adolescents regarding human papillomavirus. Rev Saude Publica. 2022 Apr 1;56:12. doi: 10.11606/s1518-8787.2022056003639. eCollection 2022.

177. Pinkas W, Jankowski M, Wierzba W. Factors Associated with Attitudes towards Preventing Head and Neck Cancer through HPV Vaccination in Poland: A Nationwide Cross-Sectional Survey in 2021. Vaccines (Basel). 2022 Apr 18;10(4):632. doi: 10.3390/vaccines10040632.

178. Kiener LM, Schwendener CL, Jafflin K, et al. Vaccine hesitancy and HPV vaccine uptake among male and female youth in Switzerland: a cross-sectional study. BMJ Open. 2022 Apr 21;12(4):e053754. doi: 10.1136/bmjopen-2021-053754.

179. M S, A V, Saurish, Amoghashree, Narayanamurthy MR, Gopi A. Did this pandemic trigger a spike in mothers' hesitancy over their children's routine immunizations? -A cross sectional study. Clin Epidemiol Glob Health. 2022 May-Jun;15:101023. doi: 10.1016/j.cegh.2022.101023. Epub 2022 Mar 18.

180. Hussein YHH, Ibrahim MH, Badran SG, Eldeeb SM. Hesitancy for influenza vaccine among healthcare workers and mothers of preschool children: A cross-sectional study in Zagazig, Egypt. J Family Community Med. 2022 May-Aug;29(2):108-116. doi: 10.4103/jfcm.jfcm_95_22. Epub 2022 May 13.

181. Winarto H, Habiburrahman M, Dorothea M, et al. Knowledge, attitudes, and practices among Indonesian urban communities regarding HPV infection, cervical cancer, and HPV vaccination. PLoS One. 2022 May 12;17(5):e0266139. doi: 10.1371/journal.pone.0266139. eCollection 2022.

182. Derdemezis C, Markozannes G, Rontogianni MO, et al. Parental Hesitancy towards the Established Childhood Vaccination Programmes in the COVID-19 Era: Assessing the Drivers of a Challenging Public Health Concern. Vaccines (Basel). 2022 May 20;10(5):814. doi: 10.3390/vaccines10050814.

183. Aimagambetova G, Babi A, Issa T, Issanov A. What Factors Are Associated with Attitudes towards HPV Vaccination among Kazakhstani Women? Exploratory Analysis of Cross-Sectional Survey Data. Vaccines (Basel). 2022 May 23;10(5):824. doi: 10.3390/vaccines10050824.

184. AlOmran HI, Al-Dosary AS, AlGhamdi FM, Alshahrani ZM, Altayar NS. Exploring parents' knowledge and attitudes towards the influenza vaccine in a rural community of Saudi Arabia. J Public Health Afr. 2022 May 24;13(1):2207. doi: 10.4081/jphia.2022.2207. eCollection 2022 May 24.

185. Mihretie GN, Liyeh TM, Ayele AD, Belay HG, Yimer TS, Miskr AD. Knowledge and willingness of parents towards child girl HPV vaccination in Debre Tabor Town, Ethiopia: a community-based cross-sectional study. Reprod Health. 2022 Jun 10;19(1):136. doi: 10.1186/s12978-022-01444-4.

186. Lai X, Li M, Hou Z, et al. Factors associated with caregivers' hesitancy to vaccinate children against influenza: A cross-sectional survey in China. Vaccine. 2022 Jun 23;40(29):3975-3983. doi: 10.1016/j.vaccine.2022.05.023. Epub 2022 May 28.

187. Mari A, Gianolio L, Edefonti V, et al. HPV Vaccination in Young Males: A Glimpse of Coverage, Parental Attitude and Need of Additional Information from Lombardy Region, Italy. Int J Environ Res Public Health. 2022 Jun 24;19(13):7763. doi: 10.3390/ijerph19137763.

188. Rositch AF, Liu T, Chao C, Moran M, Beavis AL. Levels of Parental Human Papillomavirus Vaccine Hesitancy and Their Reasons for Not Intending to Vaccinate: Insights From the 2019 National Immunization Survey-Teen. J Adolesc Health. 2022 Jul;71(1):39-46. doi: 10.1016/j.jadohealth.2022.01.223. Epub 2022 Mar 9.

189. Hou Z, Guo J, Lai X, et al. Influenza vaccination hesitancy and its determinants among elderly in China: A national cross-sectional study. Vaccine. 2022 Aug 5;40(33):4806-4815. doi: 10.1016/j.vaccine.2022.06.063. Epub 2022 Jul 1.

190. Prada-García C, Fernández-Espinilla V, Hernán-García C, et al. Attitudes, Perceptions and Practices of Influenza Vaccination in the Adult Population: Results of a Cross-Sectional Survey in Spain. Int J Environ Res Public Health. 2022 Sep 5;19(17):11139. doi: 10.3390/ijerph191711139.

191. Yakum MN, Funwie AD, Ajong AB, Tsafack M, Ze LEE, Shah Z. The burden of vaccine hesitancy for routine immunization in Yaounde-Cameroon: A cross-sectional study. PLOS Glob Public Health. 2022 Sep 26;2(9):e0001012. doi: 10.1371/journal.pgph.0001012. eCollection 2022.

192. Alnumair A, Almulifi A. Perceptions toward childhood vaccinations (side effects vs. benefits) among the parents living in Hail, Saudi Arabia. J Family Med Prim Care. 2022 Oct;11(10):6285-6290. doi: 10.4103/jfmpc.jfmpc_861_22. Epub 2022 Oct 31.

193. Jiang B, Wang Z, Jia M, et al. Awareness, knowledge and attitude toward influenza vaccination in several population groups in China: A cross-sectional study. Front Public Health. 2022 Oct 13;10:950532. doi: 10.3389/fpubh.2022.950532. eCollection 2022.

194. Li L, Ma Y, Li W, et al. Caregiver Willingness to Vaccinate Children with Pneumococcal Vaccines and to Pay in a Low-Resource Setting in China: A Cross-Sectional Study. Vaccines (Basel). 2022 Nov 10;10(11):1897. doi: 10.3390/vaccines10111897.

195. Almaghlouth AK, Bohamad AH, Alabbad RY, Alghanim JH, Alqattan DJ, Alkhalaf RA. Acceptance, Awareness, and Knowledge of Human Papillomavirus Vaccine in Eastern Province, Saudi Arabia. Cureus. 2022 Nov 22;14(11):e31809. doi: 10.7759/cureus.31809. eCollection 2022 Nov.

196. Ukumo EY, Weldehawariat FG, Dessalegn SA, Minamo DM, Weldehawaryat HN. Acceptance of Human Papillomavirus Vaccination and Associated Factors among Girls in Arba Minch Town, Southern Ethiopia, 2020. Infect Dis Obstet Gynecol. 2022 Dec 7;2022:7303801. doi: 10.1155/2022/7303801. eCollection 2022.

197. Fan J, Ye C, Wang Y, et al. Parental Seasonal Influenza Vaccine Hesitancy and Associated Factors in Shanghai, China, during the COVID-19 Pandemic: A Cross-Sectional Study. Vaccines (Basel). 2022 Dec 9;10(12):2109. doi: 10.3390/vaccines10122109.

198. Alkalash SH, Alshamrani FA, Alhashmi Alamer EH, Alrabi GM, Almazariqi FA, Shaynawy HM. Parents' Knowledge of and Attitude Toward the Human Papillomavirus Vaccine in the Western Region of Saudi Arabia. Cureus. 2022 Dec 19;14(12):e32679. doi: 10.7759/cureus.32679. eCollection 2022 Dec.

199. Al-Regaiey KA, Alshamry WS, Alqarni RA, et al. Influence of social media on parents' attitudes towards vaccine administration. Hum Vaccin Immunother. 2022 Dec 31;18(1):1872340. doi: 10.1080/21645515.2021.1872340. Epub 2021 Feb 17.

200. Babi A, Issa T, Issanov A, et al. Knowledge and attitudes of mothers toward HPV vaccination: A cross-sectional study in Kazakhstan. Womens Health (Lond). 2023 Jan-Dec;19:17455057231172355. doi: 10.1177/17455057231172355.

201. Ha Y, Lee K, Park B, Suh M, Jun JK, Choi KS. Parental intention to vaccinate daughters with the human papillomavirus vaccine in Korea: a nationwide cross-sectional survey. Epidemiol Health. 2023;45:e2023076. doi: 10.4178/epih.e2023076. Epub 2023 Aug 17.

202. Zhang Z, Shi J, Zhang X, Guo X, Yu W. Willingness of parents of 9-to-18-year-old females in China to vaccinate their daughters with HPV vaccine. Vaccine. 2023 Jan 4;41(1):130-135. doi: 10.1016/j.vaccine.2022.11.016. Epub 2022 Nov 18.

203. Gundogdu Z, Yendur Sezer O. Changing Parental Attitudes Towards Rotavirus Vaccine. Cureus. 2023 Feb 23;15(2):e35348. doi: 10.7759/cureus.35348. eCollection 2023 Feb.

204. Fadl N, Al Awaidy ST, Elshabrawy A, et al. Determinants of parental seasonal influenza vaccine hesitancy in the Eastern Mediterranean region: A cross-sectional study. Front Public Health. 2023 Mar 28;11:1132798. doi: 10.3389/fpubh.2023.1132798. eCollection 2023.

205. Choi J, Kim S, Lee SJ, Bae S, Kim S. Human Papillomavirus (HPV) Vaccination Intent among Mothers of Adolescent Sons: A National Survey on HPV Knowledge, Attitudes and Beliefs in South Korea. World J Mens Health. 2023 Apr;41(2):413-421. doi: 10.5534/wjmh.210262. Epub 2022 May 4.

206. Humble RM, Scott SD, Dubé E, Olson J, MacDonald SE. The impact of the COVID-19 pandemic on parents' perceptions and acceptance of routine childhood vaccination in Canada: A national longitudinal study. Vaccine. 2023 Jan 9;41(2):407-415. doi: 10.1016/j.vaccine.2022.11.052. Epub 2022 Nov 24.

207. Goruntla N, Akanksha K, Lalithaasudhaa K, et al. Prevalence and predictors of vaccine hesitancy among mothers of under-five children: A hospital-based cross-sectional study. J Educ Health Promot. 2023 Jan 31;12:34. doi: 10.4103/jehp.jehp_687_22. eCollection 2023.

208. Srivastav A, Lu PJ, Amaya A, et al. Prevalence of influenza-specific vaccination hesitancy among adults in the United States, 2018. Vaccine. 2023 Apr 6;41(15):2572-2581. doi: 10.1016/j.vaccine.2023.03.008. Epub 2023 Mar 10.

209. Ustuner Top F, Çevik C, Bora Güneş N. The relation between digital literacy, cyberchondria, and parents' attitudes to childhood vaccines. J Pediatr Nurs. 2023 May-Jun;70:12-19. doi: 10.1016/j.pedn.2023.01.006. Epub 2023 Feb 6.

210. Schellenberg N, Petrucka P, Dietrich Leurer M, Crizzle AM. Determinants of vaccine refusal, delay and reluctance in parents of 2-year-old children in Canada: Findings from the 2017 Childhood National Immunization Coverage Survey (cNICS). Travel Med Infect Dis. 2023 May-Jun;53:102584. doi: 10.1016/j.tmaid.2023.102584. Epub 2023 May 5.

211. Elbert B, Zainumi CM, Pujiastuti RAD, et al. Mothers' knowledge, attitude, and behavior regarding child immunization, and the association with child immunization status in Medan City during the COVID-19 pandemic. IJID Reg. 2023 May 4;8(Suppl):S22-6. doi: 10.1016/j.ijregi.2023.03.014. Online ahead of print.

212. Tekin Ç, Gökçe A, Boz G, Aslan M, Yiğit E. Reasons for parental hesitancy or refusal of childhood vaccination in Türkiye. East Mediterr Health J. 2023 May 31;29(5):343-353. doi: 10.26719/emhj.23.059.

213. Xie H, Zhu HY, Jiang NJ, Yin YN. Awareness of HPV and HPV vaccines, acceptance to vaccination and its influence factors among parents of adolescents 9 to 18 years of age in China: A cross-sectional study. J Pediatr Nurs. 2023 Jul-Aug;71:73-78. doi: 10.1016/j.pedn.2023.03.007. Epub 2023 Apr 5.

214. Alghamdi AA, Alghamdi HA. Knowledge, Attitude, and Practice of Vaccination Among Parents in Jeddah City, Saudi Arabia. Cureus. 2023 Jul 11;15(7):e41721. doi: 10.7759/cureus.41721. eCollection 2023 Jul.

215. Bitariho GK, Tuhebwe D, Tigaiza A, Nalugya A, Ssekamatte T, Kiwanuka SN. Knowledge, perceptions and uptake of human papilloma virus vaccine among adolescent girls in Kampala, Uganda; a mixed-methods school-based study. BMC Pediatr. 2023 Jul 17;23(1):368. doi: 10.1186/s12887-023-04174-z.

216. Lam EWH, Ngan HYS, Kun KY, Li DFH, Wan WY, Chan PKS. Awareness, perceptions, and acceptance of human papillomavirus vaccination among parents in Hong Kong. Hong Kong Med J. 2023 Aug;29(4):287-294. doi: 10.12809/hkmj2210470. Epub 2023 Jul 6.

217. Fadl N, Elbarazi I, Saleeb MRA, Youssef N, Shaaban R, Ghazy RM. Parental intention to vaccinate children against seasonal influenza in the Eastern Mediterranean region: A cross-sectional study using the health belief model. Hum Vaccin Immunother. 2023 Aug 1;19(2):2238513. doi: 10.1080/21645515.2023.2238513.

218. Ghazy RM, Ibrahim SA, Taha SHN, et al. Attitudes of parents towards influenza vaccine in the Eastern Mediterranean Region: A multilevel analysis. Vaccine. 2023 Aug 14;41(36):5253-5264. doi: 10.1016/j.vaccine.2023.07.005. Epub 2023 Jul 21.

219. Motilal S, Mohepath N, Moncur J, et al. Parental Knowledge, Attitudes, and Perceptions Impacting Willingness to Vaccinate Against the Human Papillomavirus in Trinidad. Cureus. 2023 Aug 16;15(8):e43581. doi: 10.7759/cureus.43581. eCollection 2023 Aug.

220. Turki YM, Alqurashi J. Knowledge, Attitudes, and Perceptions Towards Human Papillomavirus (HPV) Vaccination Among Adult Women in Primary Health Care Centers in Makkah, Saudi Arabia. Cureus. 2023 Aug 26;15(8):e44157. doi: 10.7759/cureus.44157. eCollection 2023 Aug.

221. Iova CF, Badau D, Daina MD, Șuteu CL, Daina LG. Evaluation of the Knowledge and Attitude of Adolescents Regarding the HPV Infection, HPV Vaccination and Cervical Cancer in a Region from the Northwest of Romania. Patient Prefer Adherence. 2023 Sep 8;17:2249-2262. doi: 10.2147/PPA.S421875. eCollection 2023.

222. Fuller HR, Huseth-Zosel A, Vleet BV, Hajdar M, Carson PJ. Vaccine attitudes and acceptance among older adults in North Dakota: Understanding demographic characteristic variability. Vaccine. 2023 Oct 6;41(42):6350-6358. doi: 10.1016/j.vaccine.2023.09.005. Epub 2023 Sep 9.

223. Barakat M, Abdaljaleel M, Atawneh N, et al. Pervasive Parental Hesitancy and Resistance towards Measles Rubella Vaccination in Jordan. Vaccines (Basel). 2023 Oct 31;11(11):1672. doi: 10.3390/vaccines11111672.

224. Higgins DM, Moss A, Blackwell S, O'Leary ST. The COVID-19 Pandemic and Parental Attitudes Toward Routine Childhood Vaccines. Pediatrics. 2023 Nov 1;152(5):e2023062927. doi: 10.1542/peds.2023-062927.

225. Alshehri MA, Fahim WA, Alsaigh RR. The Association Between Parents' Knowledge About Human Papillomavirus and Their Intention to Vaccinate Their Daughters: A Cross-Sectional Study. Cureus. 2023 Nov 10;15(11):e48600. doi: 10.7759/cureus.48600. eCollection 2023 Nov.

226. Iova CF, Badau D, Daina MD, Şuteu CL, Daina LG. Knowledge, Attitudes, Intentions and Vaccine Hesitancy among Postpartum Mothers in a Region from the Northwest of Romania. Vaccines (Basel). 2023 Nov 21;11(12):1736. doi: 10.3390/vaccines11121736.

227. Gari A, Ghazzawi MA, Ghazzawi SA, et al. Knowledge about cervical cancer risk factors and human papilloma virus vaccine among Saudi women of childbearing age: A community-based cross-sectional study from Saudi Arabia. Vaccine X. 2023 Jul 28;15:100361. doi: 10.1016/j.jvacx.2023.100361. eCollection 2023 Dec.

228. Hussein I, Vänskä S, Sivelä J, Leino T, Nohynek H. Factors associated with parental intention to vaccinate their child against influenza, Finland, February to March, 2022: a web-based survey. Euro Surveill. 2023 Dec;28(49):2200828. doi: 10.2807/1560-7917.ES.2023.28.49.2200828.

229. Zakhour R, Tamim H, Faytrouni F, Makki M, Hojeij R, Charafeddine L. Determinants of human papillomavirus vaccine hesitancy among Lebanese parents. PLoS One. 2023 Dec 13;18(12):e0295644. doi: 10.1371/journal.pone.0295644. eCollection 2023.

230. Tilahun S, Wondiye H, Anteneh Yigzaw Z. Mothers' intention to vaccinate their daughters against human papillomavirus in NorthWest Ethiopia, using the theory of planned behavior. Hum Vaccin Immunother. 2023 Dec 15;19(3):2288390. doi: 10.1080/21645515.2023.2288390. Epub 2023 Dec 18.

231. Alnaeem L, Alanizi S, AlQarni G, Alwadani J, Bomouzah F, Ali Z. Acceptance, Knowledge, and Attitude of Parents Toward the Human Papillomavirus Vaccine in the Eastern Region of Saudi Arabia: A Cross-Sectional Study. Cureus. 2023 Dec 29;15(12):e51293. doi: 10.7759/cureus.51293. eCollection 2023 Dec.

232. Aragaw GM, Anteneh TA, Abiy SA, Bewota MA, Aynalem GL. Parents' willingness to vaccinate their daughters with human papillomavirus vaccine and associated factors in Debretabor town, Northwest Ethiopia: A community-based cross-sectional study. Hum Vaccin Immunother. 2023 Dec 31;19(1):2176082. doi: 10.1080/21645515.2023.2176082. Epub 2023 Feb 15.

233. Didem HY, Ülfiye Ç, Miraç Ç, et al. The frequency and determinants of vaccine hesitancy among parents of preschool and kindergarten students in Edirne central district. J Pediatr Nurs. 2024 Jan-Feb;74:e38-e44. doi: 10.1016/j.pedn.2023.11.002. Epub 2023 Nov 18.

234. Fadl N, Abdelmoneim SA, Gebreal A, Youssef N, Ghazy RM. Routine childhood immunization in Sub-Saharan Africa: addressing parental vaccine hesitancy. Public Health. 2024 Jan;226:66-73. doi: 10.1016/j.puhe.2023.10.049. Epub 2023 Nov 25.

235. Čeponytė K, Narkevičiūtė E, Beržanskytė A, Burokienė S. Determinants of Parental Attitudes towards Children's Vaccination in Lithuania: An Online Survey. Acta Med Litu. 2024;31(1):42-53. doi: 10.15388/Amed.2024.31.1.7. Epub 2024 Feb 27.

236. Nguyen LTP, Phan DQ, Gyan A, Nguyen HTN, Cassell D, Huynh G. Parents' Intentions of Human Papillomavirus Vaccination for Students in Vietnam: A Cross-Sectional Study. Cancer Control. 2024 Jan-Dec;31:10732748241284907. doi: 10.1177/10732748241284907.

237. Hussein I, Vänskä S, Sivelä J, Leino T, Nohynek H. Factors associated with parental Human Papillomavirus (HPV) vaccination intention of daughter: A national survey in Finland. Vaccine. 2024 Jan 25;42(3):701-712. doi: 10.1016/j.vaccine.2023.12.026. Epub 2024 Jan 3.

238. Knijff M, van Lier A, Boer M, de Vries M, Hament JM, de Melker HE. Parental intention, attitudes, beliefs, trust and deliberation towards childhood vaccination in the Netherlands in 2022: Indications of change compared to 2013. Vaccine. 2024 Feb 6;42(4):801-811. doi: 10.1016/j.vaccine.2023.12.080. Epub 2024 Jan 12.

239. Tsige AW, Ayenew KD, Ayele SG. Assessment of knowledge and perceptions of human papillomavirus vaccine and its determinants among women who have eligible daughters in Debre Berhan City, Ethiopia: a cross-sectional study. Front Oncol. 2024 Mar 18;14:1348288. doi: 10.3389/fonc.2024.1348288. eCollection 2024.

240. Elissa N, Charbel H, Marly A, Ingrid N, Nadine S, Rachel A. Knowledge and perception of HPV vaccination among Lebanese mothers of children between nine and 17 years old. Reprod Health. 2024 Mar 27;21(1):40. doi: 10.1186/s12978-024-01764-7.

241. Wang S, Ren W, Zhang B, et al. Knowledge, Attitude, and Uptake of Human Papillomavirus (HPV) Vaccination among Chinese Female Adults: A National Cross-sectional Web-Based Survey Based on a Large E-commerce Platform. Matern Child Health J. 2024 Apr;28(4):746-757. doi: 10.1007/s10995-023-03888-x. Epub 2024 Feb 9.

242. Truong BQQ, Ong KIC, Shibanuma A, Kiriya J, Jimba M. Adaptation and application of the Parent Attitudes About Childhood Vaccines survey tool in the Vietnamese language: a cross-sectional study. BMC Public Health. 2024 Apr 2;24(1):946. doi: 10.1186/s12889-024-18389-x.

243. Ahmed WS, Halboup AM, Alshargabi A, Al-Mohamadi A, Al-Ashbat YK, Al-Jamei S. Attitudes, motivators, and barriers toward influenza vaccination for children: a study from a conflict-ridden country. Confl Health. 2024 Apr 3;18(1):26. doi: 10.1186/s13031-024-00590-9.

244. Mbonigaba E, Yu F, Reñosa MDC, et al. Knowledge and trust of mothers regarding childhood vaccination in Rwanda. BMC Public Health. 2024 Apr 17;24(1):1067. doi: 10.1186/s12889-024-18547-1.

245. Pergialiotis V, Papageorgiou D, Douligeris A, et al. Awareness, knowledge and attitudes of human papillomavirus infection, screening and vaccination: a survey study in Greece. Arch Gynecol Obstet. 2024 May;309(5):2031-2040. doi: 10.1007/s00404-024-07398-1. Epub 2024 Mar 8.

246. Alherz FA, Alamri AA, Aljbreen A, Alwallan N. Knowledge of cervical cancer, human papillomavirus (HPV), and acceptance of the HPV vaccine among parents of daughters in Riyadh, Saudi Arabia. J Infect Public Health. 2024 May;17(5):789-794. doi: 10.1016/j.jiph.2024.03.014. Epub 2024 Mar 15.

247. Alharthi TS, Alqahtani RK, Alghamdi M, et al. Awareness and Attitudes Among Parents of Females Aged 9-26 in Saudi Arabia Regarding Human Papillomavirus Vaccination. Cureus. 2024 Jun 16;16(6):e62470. doi: 10.7759/cureus.62470. eCollection 2024 Jun.

248. Al-Rashdan RA, Ta'an W, Mukattash T, Williams B. Maternal skepticism regarding children's vaccination in Jordan: Investigating the knowledge, attitude, and adherence. PLoS One. 2024 Jul 10;19(7):e0305209. doi: 10.1371/journal.pone.0305209. eCollection 2024.

249. Al Saedi RA, Al Daajani MM, Alghamdi SA. Parental Awareness, Attitude, and Acceptability of the Human Papillomavirus Vaccine in Jeddah, Saudi Arabia: An Analytical Cross-Sectional Study. Cureus. 2024 Jul 27;16(7):e65536. doi: 10.7759/cureus.65536. eCollection 2024 Jul.

250. Altom F, Khawaji NY, Almalki MM, Almohammadi WA, Al-Enezi HS, Al-Khalil SY. Knowledge, Attitude, and Perception Regarding the Human Papillomavirus (HPV) Vaccine Among Parents at Al-Madinah Al-Munawwar: A Cross-Sectional Study. Cureus. 2024 Jul 31;16(7):e65850. doi: 10.7759/cureus.65850. eCollection 2024 Jul.

251. Gao S, Li Y, Wang X, Li S, Chen M, Yue B. Vaccine literacy, vaccination intention, and their correlation among adults in Mainland China: a cross-sectional study. J Health Popul Nutr. 2024 Aug 10;43(1):122. doi: 10.1186/s41043-024-00602-7.

252. Al-Iede M, Aljahalin M, Fashho E, et al. Hesitancy toward Childhood and Influenza Vaccines: Experiences from Highly Educated Jordanian Parents. Vaccines (Basel). 2024 Aug 22;12(8):945. doi: 10.3390/vaccines12080945.

253. Barqawi HJ, Samara KA, Kannas SM, et al. Vaccine practices, literacy, and hesitancy among parents in the United Arab Emirates. PLoS One. 2024 Aug 27;19(8):e0307020. doi: 10.1371/journal.pone.0307020. eCollection 2024.

254. Kiran KA, Kujur A, Kumar D, et al. Are we really hesitant toward routine immunization: Findings from a cross-sectional study in urban area in the tribal dominant state of India. J Family Med Prim Care. 2024 Sep;13(9):4001-4007. doi: 10.4103/jfmpc.jfmpc_423_24. Epub 2024 Sep 11.

255. Bayram Değer V, Yiğitalp G. Level of hesitation of parents about childhood vaccines and affecting factors: a cross-sectional study in Turkey. BMC Public Health. 2024 Sep 13;24(1):2494. doi: 10.1186/s12889-024-19961-1.

256. Huang Y, Ling J, Zhao X, et al. Are HPV Vaccines Well Accepted among Parents of Adolescent Girls in China? Trends, Obstacles, and Practical Implications for Further Interventions: A Five-Year Follow-Up Study. Vaccines (Basel). 2024 Sep 19;12(9):1073. doi: 10.3390/vaccines12091073.

257. Chu JKP, Sing CW, Li Y, Wong PH, So EYT, Wong ICK. Factors affecting human papillomavirus vaccine acceptance among parents of Primary 4 to 6 boys and girls in Hong Kong. Hong Kong Med J. 2024 Oct;30(5):386-399. doi: 10.12809/hkmj2311144.

258. Xu J, Lu J, He Q, et al. Knowledge, Attitudes, and Practices towards Influenza Vaccine among Guangzhou Residents: A Cross-Sectional Study. Vaccines (Basel). 2024 Oct 14;12(10):1169. doi: 10.3390/vaccines12101169.

259. Negesse Simegn Y, Wossen Samuel A, Gebresilasie Kelelew R, Mohammed LA, Liyew AD, Abate Belay M. Knowledge and acceptability of the human papillomavirus vaccine and associated factors among adolescent girls in public primary schools in Dessie Town, South Wollo, Northeast Ethiopia, 2020: A cross-sectional study. BMJ Open. 2024 Oct 29;14(10):e083397. doi: 10.1136/bmjopen-2023-083397.

260. Dyda A, Lawford H, Lau CL, Sampson K. Investigating behaviours and attitudes regarding recommended vaccination in adults 50 years and above in Australia. Aust J Prim Health. 2024 Nov;30:PY24055. doi: 10.1071/PY24055.

261. Hawlader MDH, Eva FN, Khan MAS, et al. Acceptance of Human Papillomavirus (HPV) vaccine among the parents of eligible daughters (9-15 years) in Bangladesh: A nationwide study using Health Belief Model. PLoS One. 2024 Nov 8;19(11):e0310779. doi: 10.1371/journal.pone.0310779. eCollection 2024.

262. Walters M, Rhodes A, Hoq M, Measey MA. Caregiver's intention and uptake of influenza vaccination for children in Australia before and after introduction of free vaccination. Public Health. 2024 Dec;237:94-96. doi: 10.1016/j.puhe.2024.08.022. Epub 2024 Sep 30.

263. Yusuf KK, Olorunsaiye CZ, Gadanya MA, Ouedraogo S, Abdullahi AA, Salihu HM. HPV vaccine hesitancy among parents and caregivers of adolescents in Northern Nigeria. Vaccine X. 2024 Nov 23;21:100591. doi: 10.1016/j.jvacx.2024.100591. eCollection 2024 Dec.

264. Bai N, Wang Q, Shao J, et al. HPV vaccination intention among guardians of female secondary school students in Chongqing, China. Hum Vaccin Immunother. 2024 Dec 31;20(1):2381293. doi: 10.1080/21645515.2024.2381293. Epub 2024 Aug 14.
