# Supplementary material for: Mapping the Global Landscape of Vaccine Acceptance Among the General Population from 2009 to 2024: A Systematic Review Across COVID-19 Pandemic Phases, Vaccine Categories, and Economic Strata
Source: Vaccines (Basel). 2026 Jul 29;14(8):663. doi: 10.3390/vaccines14080663 (PMC13517538; doi:10.3390/vaccines14080663)
Supplement: Supplementary file 1 [file vaccines-14-00663-s001.zip › File S4 - Funnel plot Funnel-plot and linear regression test of asymmetry.pdf]

File S4 - Funnel-plot and linear regression test of asymmetry

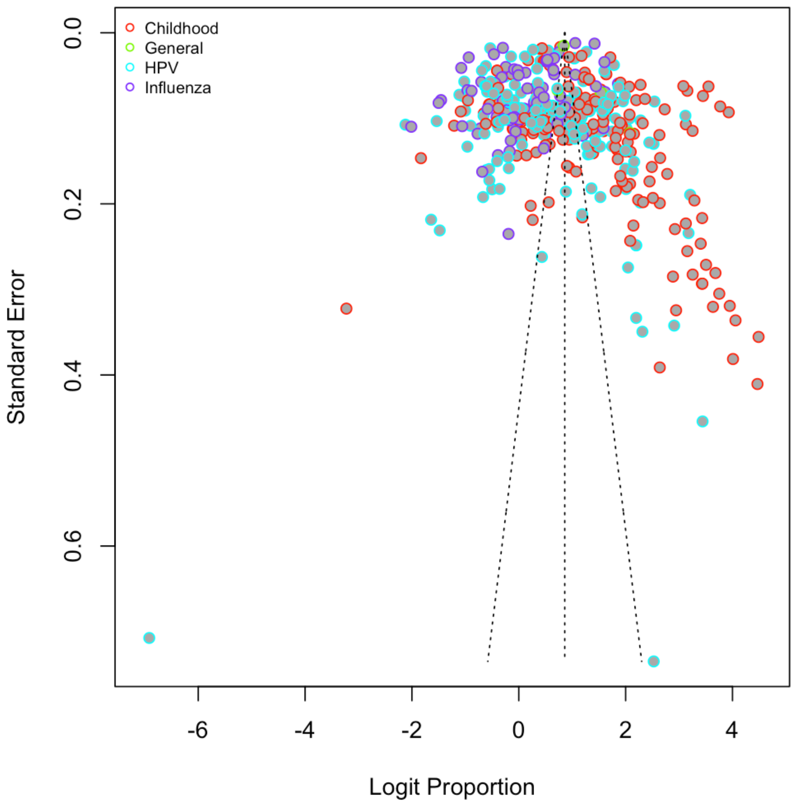

Figure: Funnel-plot assessing publication bias

Publication bias was assessed using Egger's linear regression test of funnel-plot asymmetry (Egger et al., 1997), with standard error as the predictor and inverse-variance weighting. The test showed no statistically significant evidence of funnel-plot asymmetry (bias estimate = 2.052, SE = 1.11; t = 1.85, df = 405, p = 0.065). Given the substantial between-study heterogeneity ($\tau^2 \approx 200.9$) inherent to a synthesis spanning multiple vaccine categories, pandemic phases, and economic strata, any residual asymmetry is more plausibly attributable to true heterogeneity than to selective publication. The funnel plot above (Appendix 4, Supplementary materials) supports a broadly symmetrical distribution of effect estimates.

Reference:

Egger M., Davey Smith G., Schneider M., Minder C. Bias in meta-analysis detected by a simple, graphical test. BMJ. 1997;315(7109):629-34. doi: https://doi.org/10.1136/bmj.315.7109.629
