# Supplementary material for: Mapping the Global Landscape of Vaccine Acceptance Among the General Population from 2009 to 2024: A Systematic Review Across COVID-19 Pandemic Phases, Vaccine Categories, and Economic Strata
Source: Vaccines (Basel). 2026 Jul 29;14(8):663. doi: 10.3390/vaccines14080663 (PMC13517538; doi:10.3390/vaccines14080663)
Supplement: Supplementary file 1 [file vaccines-14-00663-s001.zip › File S5 - Critical Appraisal of Included Studies.pdf]

File S5 - Critical Appraisal of Included Studies

A total of 264 studies were appraised for methodological quality using the JBI Critical Appraisal Checklist for Studies Reporting Prevalence Data. The overall methodological quality varied across the nine domains as shown in figure below.

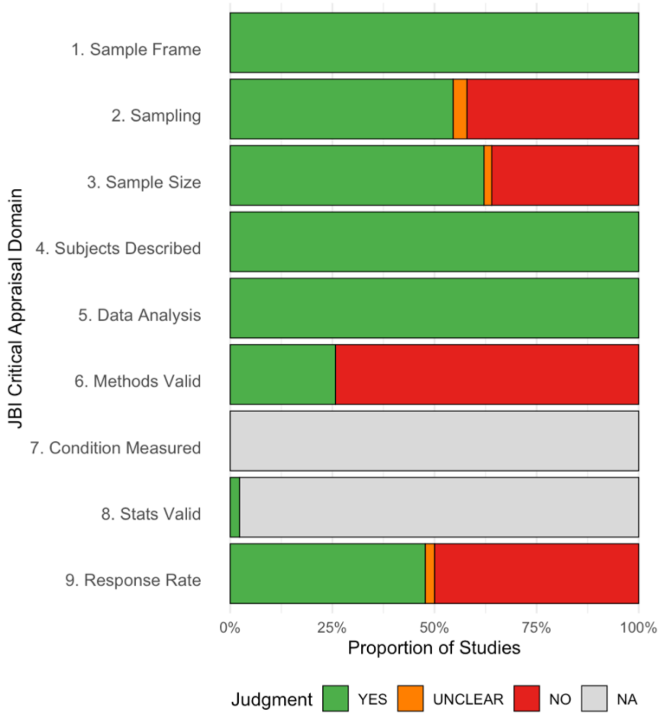

Figure: Critical Appraisal of 264 studies Included in the Systematic Review

Sample Frame and Study Subjects (Questions 1 & 4): All 264 included studies (100%) utilized an appropriate sampling frame and adequately described the study subjects and setting. This complete adherence is a direct result of the author's inclusion criteria, which specifically targeted the general public without any comorbidities or the minority groups.

Sampling Method (Question 2): Appropriate probabilistic sampling methods were clearly identified in 54.55% of the studies. Studies relying on convenience sampling, snowball sampling, or those failing to provide a clear description of their recruitment methodology were assessed as No or unclear for this domain.

Sample Size (Question 3): An adequate sample size was confirmed in 62.12% of the studies. A "Yes" judgment was assigned strictly to studies that either reported a sample size calculation or effectively surveyed the entirety of a defined target population within the study timeframe.

Data Analysis Coverage (Question 5): Given that the primary objective of this meta-analysis targets the general population rather than specific subgroup comparisons, data analysis was considered to have sufficient coverage of the identified sample across all studies (100%).

Validity of Methods (Question 6): Valid methods for the identification of the condition were observed in only 25.76% of the literature. Because there is no universally standardized diagnostic tool for measuring vaccine hesitancy that can be applicable across all vaccines and contexts, only studies utilizing measurement instruments that had been previously published and validated were appraised as "Yes". However, substantial heterogeneity in the specific tools utilized was still noted among these studies.

Standardized Measurement (Question 7): This domain was deemed "Not Applicable" (NA) across the board. Vaccine hesitancy is inherently a subjective, self-reported psychological construct; therefore, assessing whether the condition was measured in an objective, standardized, and clinically reliable way for all participants (e.g., via trained clinical observers) does not apply to this systemic review and meta-analysis.

Appropriateness of Statistical Analysis (Question 8): This domain was rated as "Not Applicable" for the vast majority of the included studies. The primary objective for data extraction in this meta-analysis was to obtain the raw numerator and denominator (the population size and the specific number of hesitant/ acceptance cases), rather than relying on the authors' reported prevalence estimates. Only six studies (2.27%) that explicitly reported prevalence data with 95% confidence intervals intended for broad country- or county-level extrapolation were appraised as "Yes".

Response Rate (Question 9): An adequate response rate, or the appropriate management and discussion of a low response rate, was recorded in 47.73% of the appraised studies.

Reference:

Munn Z., Moola S., Lisy K., Riitano D., Tufanaru C. Chapter 5: Systematic reviews of prevalence and incidence. In: Aromataris E, Munn Z (Editors). JBI Manual for Evidence Synthesis. JBI, 2020. Available at: https://jbi.global/critical-appraisal-tools
